# Fluorescence-Based Quantitative Synapse Analysis for Cell Type-Specific Connectomics

**DOI:** 10.1523/ENEURO.0193-19.2019

**Published:** 2019-10-23

**Authors:** Dika A. Kuljis, Eunsol Park, Cheryl A. Telmer, Jiseok Lee, Daniel S. Ackerman, Marcel P. Bruchez, Alison L. Barth

**Affiliations:** Department of Biological Sciences, Carnegie Mellon University, Pittsburgh, PA 15213

**Keywords:** barrel cortex, PV, pyramidal cell, SST, VIP, anatomy

## Abstract

Anatomical methods for determining cell type-specific connectivity are essential to inspire and constrain our understanding of neural circuit function. We developed genetically-encoded reagents for fluorescence-synapse labeling and connectivity analysis in brain tissue, using a fluorogen-activating protein (FAP)-coupled or YFP-coupled, postsynaptically-localized neuroligin-1 (NL-1) targeting sequence (FAP/YFPpost). FAPpost expression did not alter mEPSC or mIPSC properties. Sparse AAV-mediated expression of FAP/YFPpost with the cell-filling, red fluorophore dTomato (dTom) enabled high-throughput, compartment-specific detection of putative synapses across diverse neuron types in mouse somatosensory cortex. We took advantage of the bright, far-red emission of FAPpost puncta for multichannel fluorescence alignment of dendrites, FAPpost puncta, and presynaptic neurites in transgenic mice with saturated labeling of parvalbumin (PV), somatostatin (SST), or vasoactive intestinal peptide (VIP)-expressing neurons using Cre-reporter driven expression of YFP. Subtype-specific inhibitory connectivity onto layer 2/3 (L2/3) neocortical pyramidal (Pyr) neurons was assessed using automated puncta detection and neurite apposition. Quantitative and compartment-specific comparisons show that PV inputs are the predominant source of inhibition at both the soma and the dendrites and were particularly concentrated at the primary apical dendrite. SST inputs were interleaved with PV inputs at all secondary-order and higher-order dendritic branches. These fluorescence-based synapse labeling reagents can facilitate large-scale and cell-type specific quantitation of changes in synaptic connectivity across development, learning, and disease states.

## Significance Statement

High-throughput quantitation of synapse number and distribution can reveal principles of circuit function and their adaptive or pathologic alterations. Molecular genetic, fluorescence-based approaches targeted to discrete cell types can enable automated detection and quantification of input-specific synapses in complex brain tissues. In addition, these tools present a low barrier to use within the neuroscience community through volumetric confocal analysis of tissue specimens. Here we evaluate inhibitory synapse distribution across layer 2/3 (L2/3) pyramidal (Pyr) neurons using postsynaptic expression of a previously characterized, neuroligin-based construct. We find that inhibitory inputs from fluorescently-labeled parvalbumin (PV) and somatostatin (SST) neurons are intermingled across the proximal dendrites, and that inputs from vasoactive intestinal peptide (VIP) neurons are rare for L2/3 Pyr neurons.

## Introduction

The organization, number, and input identity of synapses onto a cell are critical determinants of neuronal activity. Although electrophysiological analyses of synaptic properties have provided a rich framework to build and test hypotheses about neural computations during sensation and behavior, these analyses cannot reveal broader principles of synaptic distribution across the neuron. Since alterations to synaptic function in select circuits and cell types are associated with autism, intellectual disability, psychiatric, and neurologic disease (Bayés et al., 2011; Südhof, 2017), quantitative metrics about synaptic location, size, and input specificity are likely to provide key insights into how neural circuits are related to disease pathology.

Electron microscopy (EM) provides nanometer resolution for ultrastructural identification of synaptic contacts and has been employed for brain-area and cell-type quantitative analysis (Bock et al., 2011; Briggman et al., 2011; Kim et al., 2014; Chandrasekaran et al., 2015); however, EM is hampered by technical demands of sample preparation, imaging time, data storage, and labor-intensive analysis that make comparisons across multiple individuals or conditions difficult. Recent studies have attempted to use EM for quantitative analysis of synapse organization between defined presynaptic and postsynaptic partners, but these computationally-intensive approaches are difficult to adopt and scale for broad use (Kubota et al., 2015; Glausier et al., 2017; Kornfeld et al., 2017; Vishwanathan et al., 2017). Fluorescence-based microscopy methods are an attractive alternative to EM, because light microscopy facilitates faster acquisition of larger tissue volumes and enables use of spectrally distinct, genetically encoded fluorophores for discrimination of molecularly diverse cells and synapse types.

There has been great interest in developing tools and methodologies for synapse labeling using molecular, genetic, or histochemical techniques for light microscopy, including GFP-tagging synaptic molecules, GFP reconstitution across synaptic partners (GRASP), and array tomography (Micheva and Smith, 2007; Kim et al., 2011; Chen et al., 2012; Gross et al., 2013; Fortin et al., 2014; Martell et al., 2016; Villa et al., 2016; Kinoshita et al., 2019). Fluorescence-based, sparse labeling of postsynaptic neurons in intact brain tissue has been especially helpful in this regard, as it reduces the analysis bottleneck that arises from broadscale immunohistochemical labeling of synapses from neurons intermingled in the analysis volume. However, high-throughput/volumetric synaptic analysis for individual neurons has not yet become routine, possibly due to low signal-to-noise and synaptogenesis or abnormal synapse stabilization associated with overexpression of synaptic tags (El-Husseini et al., 2000; Kim et al., 2011; Tsetsenis et al., 2014; Martell et al., 2016).

We sought to develop molecular genetic tools for comprehensive fluorescence labeling of postsynaptic sites across an individual neuron, in a complex tissue environment. We employed fluorogen-activating protein (FAP), a modified antibody fragment that emits in the far red on binding of a small molecule ligand, a derivative of malachite green (MG; Szent-Gyorgyi et al., 2013), targeted to postsynaptic sites using the well-validated postsynaptic tag derived from the transmembrane and cytoplasmic region of mouse neuroligin-1 (NL-1, FAPpost; Kim et al., 2011; Druckmann et al., 2014; Kwon et al., 2018).

Sparse, virus-mediated coexpression of FAPpost with the cell-filling fluorophore dTomato (dTom) showed broad, punctate labeling across distinct pyramidal (Pyr) cell compartments. Aided by automated image analysis, we quantitatively evaluated the distribution of inhibitory synapses identified from analysis of >90,000 synaptic puncta in neocortical Pyr neurons in superficial layers of somatosensory cortex. Using comprehensive fluorescence-labeling of cell type specific neurites in parvalbumin (PV), somatostatin (SST), and vasoactive intestinal peptide (VIP) Cre-driver transgenic mice allowed us to align FAPpost puncta to quantify inhibitory inputs for layer 2/3 (L2/3) Pyr neurons. This quantitative analysis revealed that PV inputs dominated the soma and the synapse-dense 1° apical dendrite, and that PV inputs had a moderately higher density than SST inputs across all L2/3 dendrites. VIP neurons only sparsely innervated L2/3 Pyr neurons. These studies help establish a framework for a high-throughput analysis of synapse organization in brain tissue during health and disease.

## Materials and Methods

All experimental procedures were conducted in accordance with the National Institutes of Health guidelines and were approved by the Institutional Animal Care and Use Committee at Carnegie Mellon University.

### Construct design

#### FAPpost cloning

To make the plasmid for packaging into AAV, post-mGRASP from Addgene (#34912, paavCAG-post-mGRASP-2A-dTom) was modified by annealing oligos and inserting into BamHI and XhoI digested backbone to introduce an AgeI site (PostBamXhoF 5’GATCC CTT ACCGGT ATC TTA C and PostBamXhoR 5’ TCGAG TAA GAT ACCGGT AAG G). PCR was used to produce the Igkappa leader sequence, cmyc epitope and dL5** FAP (Szent-Gyorgyi et al., 2008, 2013; Telmer et al., 2015) for introduction into the BamHI and AgeI of the modified backbone (BamKappaF 5′-TATATA GGATCC ggcttggggatatccaccatgg and dL5AgeSfiR 5′-TATATA ACCGGT ACCTCC ggccagaccggccgc GGAGAG). The BamHI/HindIII fragment was moved to create pENN.AAV.hSyn.kappa.myc.dL5.POSTsyn.T2A.dTom.WPRE.BGH (Addgene FAPpost plasmid RRID: addgene_105981). AAV1 serotype was produced by Penn Vector Core.

#### Fl-YFPpost and fl-FAPpost cloning

For Cre-inducible expression, the kappa.myc.dL5.POSTsyn.T2A.dTom region was PCR amplified with primers containing BsrG1 and KpnI restriction sites (partial KpnI digestion was required) and ligated into digested pAAV-FLEX (fl; generous gift from Oliver Schluter) to produce pAAV-FLEX-hSyn-kappa-myc-dL5-POSTsyn-T2A.dTom-WPRE-SV40. PCR amplification was used to generate the SYFP2 (YFP; Kremers et al., 2006) coding fragment (iGEM BBa_K864100) that was then SfiI digested to replace the FAP in the pAAV-FLEX resulting in pAAV-FLEX-hSyn-kappa-myc-dL5-POSTsyn-T2A-dTom-WPRE-SV40 (Addgene fl-FAPpost plasmid RRID: addgene_105982; Addgene fl-YFPpost plasmid RRID: addgene_105983). Constructs were packaged into AAV1 and produced by Penn Vector Core.

#### Animals

Experiments were performed on wild-type and transgenic reporter male and female mice on a C57BL6J background (Table 1). Cre recombinase lines used included Emx1-IRES-Cre (The Jackson Laboratory stock #005628), Pvalb-2A-Cre (The Jackson Laboratory stock #008069; Hippenmeyer et al., 2005), SST-IRES-Cre (The Jackson Laboratory stock #013044; Taniguchi et al., 2011), and VIP-IRES-Cre (The Jackson Laboratory stock #010908; Taniguchi et al., 2011). Homozygous Cre-expressing mice were mated with homozygous Ai3 mice (The Jackson Laboratory stock #007903) to create heterozygous transgenic mice with eYFP- (YFP)-labeled SST, PV, or VIP interneurons. Pyr cells from at least three mice from each line were used to characterize FAPpost expression patterns.

**Table 1:** Experimental metadata

Cell type	Construct	Animal ID	Sex	Cell ID	Genotype	Age	DPI	*n*	Mean ± SEM*
Pyr	FAPpost	BZS1	M	5, 11, 12	Ai3xVIP-Cre	24	9	3	3.40 ± 0.32
		BPP8	F	1, 11	Ai3xPV-Cre	24	11	2	1.38 ± 0.38
		BZS3	M	2, 3, 4, 5	Ai3xVIP-Cre	25	10	4	2.93 ± 0.13
		BQW1	M	11	Ai3xSST-Cre	25	11	1	2.15 ± NA
		BQW4	F	6, 10, 11, 12	Ai3xSST-Cre	25	11	4	2.09 ± 0.17
		BXT6	M	1, 2	Ai3xSST-Cre	25	8	2	2.08 ± 0.14
		BYS1	F	1, 3	Ai3xSST-Cre	26	9	2	1.32 ± 0.36
		BLN11	F	17	Ai3xPV-Cre	27	15	1	2.78 ± NA
		BFE4	M	2, 10	WT	27	13	2	2.18 ± 0.02
		BLN7	F	6, 7	Ai3xPV-Cre	27	15	2	1.67 ± 0.03
		BLW4	F	43, 45	Ai3xVIP-Cre	28	14	2	2.53 ± 0.44
		CHI3	F	3, 4, 5, 9	Ai3xPV-Cre	28	12	4	2.17 ± 0.21
	YFPpost	CPV1	F	1, 3, 4	Emx1-Cre	22	7	3	2.77 ± 0.15
		CNH4	M	1, 2, 4, 5, 6, 7, 8	Emx1-Cre	24	10	7	2.98 ± 0.24
		CNH5	F	2, 3, 4, 5, 6	Emx1-Cre	24	10	5	2.34 ± 0.06
		CPW1	F	1, 2, 3, 4, 5, 6	Emx1-Cre	27	10	6	3.21 ± 0.29

*Animal average (dendritic density across cells, excluding 1° apical dendrite and soma).

#### Virus injection surgery

FAPpost virus (0.4 μl) was stereotaxically injected into barrel cortex through a small craniotomy (bregma –0.9, lateral 3.00, depth 0.5 mm) in isoflurane-anaesthetized mice aged postnatal day (P)12–P17 using a Hamilton syringe (Hamilton), Stoelting infusion pump (Stoelting; model #53210), and custom injection cannulas (Plastics One). Mice were treated once with ketofen (5 mg/kg, Sigma-Aldrich), then allowed to recover in their home cage until weaning (P21), when they were moved to a new cage with their littermates.

#### Fixed tissue preparation and immunohistochemistry

Seven to 15 d following virus injection, animals were anesthetized with isoflurane and transcardially perfused at mid-day using 20 ml PBS (pH 7.4) followed by 20 ml 4% paraformaldehyde in PBS (PFA; pH 7.4). Brains were removed, and postfixed overnight at 4°C in 4% PFA before transfer into 30% sucrose cryoprotectant. After osmotic equilibration brains were sectioned (50-μm-thick section) using a freezing-microtome.

Free-floating brain sections containing dTom-expressing cells were washed using PBS before 30-min room temperature incubation with MG dye (300 nM in PBS; Pratt et al., 2017). MG-dyed sections were then rinsed with PBS before mounting on glass microscope slides with Vectashield fluorescent mounting media (Vector Lab). Before MG dye application, a subset of brain sections underwent immunofluorescence staining. Brain sections from PV-Cre, SST-Cre, or VIP-Cre x Ai3 for saturated cell type-specific YFP labeling underwent GFP immunofluorescence staining to enhance YFP signal. These sections were first blocked (10% NGS, 0.1% Triton X-100, and 0.1 M PBS), and incubated for 48 h at 4°C with anti-GFP primary antibody (1:2000 dilution in blocking solution; Abcam AB13970). Sections were rinsed with PBS, then incubated with Alexa Fluor 488 secondary antibody (1:500, in blocking solution; Invitrogen A-11039). In the same manner, a subset of PV-Cre x Ai3 brain sections underwent bassoon immunofluorescence staining to visualize presynaptic release sites. These sections were blocked (10% DS, 0.2% Triton X-100, and 0.1 M PBS), then incubated overnight at 4°C with mouse anti-bassoon primary antibody (1:1500 in blocking solution; Enzo Life Sciences Assay Design VAM-PS003). Slices were rinsed with 0.2% PBST then incubated with Alexa Fluor 405 anti-mouse secondary antibody (1:500 dilution in blocking solution; Invitrogen A-31553).

#### Confocal imaging

FAPpost expression in L2/3 (∼200–300 μm below the pial surface) of the S1 barrelfield (S1BF) was confirmed by the presence of layer 4 barrels. Pyr cells were identified using morphologic criteria, including the presence of a thick apical dendrite oriented toward the pial surface, Pyr-shaped cell body, laterally projecting basal dendrites, a descending axon identified by its narrow diameter, and the ubiquitous presence of dendritic spines, particularly on higher-order branches. Only isolated Pyr cells (typically at the edge of the viral transduction zone) that exhibited dendritic dTom as well as punctate FAP signal were selected for imaging and quantitation. FAPpost and dTom expression were not always positively correlated, an effect that was unexpected given the construct design. dTom-expressing neurons that did not exhibit membrane-localized FAP fluorescence, or showed diffuse and low-intensity signal were excluded from analysis. Analyzed cells showed no significant relationship between FAPpost puncta intensity and calculated puncta density along the dendrite. In almost all cases, selected cells included the entire soma in the image dataset. Because cortical dendrites are >200 μm long and could lie outside the imaged area, only a fraction of the dendritic arbor was collected and analyzed.

Sections were observed under a LSM 880 AxioObserver Microscope (Zeiss), using 63× oil-immersion objective lens (Plan-Apochromat, 1.40 Oil DIC M27) with the pinhole set at 1.0 Airy disk unit. Maximum image size was 1024 × 1024 pixels. Zoom factor was set to 1, corresponding to a voxel dimension of 0.13 × 0.13 × 0.32 μm in *x*, *y*, and *z* directions. Selected cell bodies were centered in the field of view (135 × 135 μm). Up to 100 images with a Z-interval of 0.32 μm and 50% overlap between optical sections were acquired per stack. Fluorescence acquisition settings were as follows: Alexa Fluor 405 (excitation λ405, emission λ452, detection λ406–488), Alexa Fluor 488 (excitation λ488, emission λ504, detection λ490–517), YFP (excitation λ514, emission λ535, detection λ517–535), dTom (excitation λ561, emission λ579, detection λ561–597), and MG/FAP (excitation λ633, emission λ668, detection λ641–695). Optimal laser intensities for each channel were set for each cell independently, and images were collected to avoid pixel saturation. Well-isolated cells of interest were centered in the image frame and the Z-stack dimensions were set manually by tracking dTom-labeled dendrites. Z-stacks typically ranged from 30 to 40 μm for a given neuron. For experiments assessing bassoon immunofluorescence alignment with YFP-expressing PV neurites and FAPpost puncta on soma of transduced cells, image size was 1912 × 1912 pixels with a zoom factor was set to 2, corresponding to a voxel dimension of 0.05 × 0.05 × 0.32 μm in *x*, *y*, and *z* directions. The total Z-stack depth typically ranged between 10 and 15 μm starting from the brain section surface, where bassoon antibody penetration was most complete.

#### Cranial window construction for in vivo imaging

One week after virus injection, mice were isoflurane anesthetized and head fixed using a custom-made nose clamp. Eyes were covered with ointment, hair was removed with Nair, and scalp was disinfected with povidone iodine. Scalp and periosteum were removed, and skull surface roughened by scraping with a slowly rotating dental drill. A thin layer of Krazyglue was applied to the skull before a custom-made head bracket was attached in the right hemisphere using Krazyglue and dental cement (Lang Dental, 1223PNK). The skull was carefully thinned around a 3-mm diameter circle centered above the left hemisphere S1BF using a dental drill (Dentsply, 780044). After extensive thinning, the loose bone flap was detached using a microforcep. A glass window composed of 3 mm diameter glass (Harvard Apparatus, 64-0720) attached to a 5 mm diameter glass (Harvard Apparatus, 64-0700) was mounted above the exposed brain immediately before acute imaging. The window was sealed with 3M^TM^ Vetbond^TM^. A chamber wall was built around the window with dental cement. Ketoprofen (3 mg/kg) was given subcutaneously.

#### Two-photon (2P) imaging

Mice were anesthetized with 1.5% isoflurane and mounted under a Femtonics FEMTO_2_D microscope. Layer 1/2 dendrites expressing dTom and YFPpost were visualized under a 63× objective using 950-nm excitation (Spectra-Physics Mai Tai HP) with simultaneous detection of dTom and YFPpost using red and green PMTs, respectively. Single-plane 60 × 60 μm (1000 × 1000 pixel) linescan (15× averaging) images were acquired using MES software (Femtonics, v.5.2878). The raw intensity matrix for each channel was converted to a grayscale image in MATLAB (MathWorks, R2017a). Channels were overlaid and brightness/contrast adjusted using Photoshop 6.0 (Adobe).

#### Electrophysiology

FAPpost-injected mice were sacrificed at age P20–P25 by brief isoflurane anesthesia and decapitation. Coronal slices (350 μm thick) were prepared in regular ice-cold artificial cerebrospinal fluid (ACSF) composed of the following: 119 mM NaCl, 2.5 mM KCl, 1 mM NaH_2_PO_4_, 26.2 mM NaHCO_3_, 11 mM glucose, 1.3 mM MgSO_4_, and 2.5 mM CaCl_2_ equilibrated with 95%/5% O_2_/CO_2_. Slices recovered in the dark at room temperature for 60 min before transfer to an electrophysiology rig where they were perfused with ACSF containing 1 μM tetrodotoxin (Tocris) to silence spontaneous activity. The injection site was identified by dTom fluorescent cell bodies using an Olympus light microscope (BX51WI). Pyr-targeted recordings (four to five animals per group) were done in the absence of MG dye, since we were interested in whether *in vivo* expression of the FAPpost construct would influence synaptic function and MG was never applied before tissue fixation for anatomic analysis. Borosilicate glass electrode resistance was 4–8 MΩ. Electrode internal solution was composed of the following: 130 mM cesium gluconate, 20 mM HEPES, 0.4 mM EGTA, 2.8 mM NaCl, 5 mM tetraethylammonium chloride (TEA-Cl), 4 mM Mg-ATP, and 0.3 mM Na-GTP; pH 7.25–7.30, 280–290 mOsm. Trace amounts of Alexa Fluor 488 (Invitrogen A10436) were included in the internal solution to confirm that targeted cells had Pyr-like morphologies. Electrophysiological data were acquired using a Multiclamp 700B amplifier (Molecular Devices) and a National Instruments acquisition interface (National Instruments). The data were filtered at 3 kHz, digitized at 10 kHz and collected by Igor Pro 6.0 (Wavemetrics). After forming a GΩ seal, negative pressure was applied to the cell to enter whole-cell mode, and following 2- to 3-min acclimation time, miniature EPSCs (mEPSCs) were collected at –70 mV holding potential for 5 min. Holding potential was slowly raised to 0 mV over an additional minute, and following 1 min acclimation time, miniature IPSCs (mIPSCs) were then collected. Traces were analyzed using MiniAnalysis (Synaptosoft Inc.), with a 7-pA minimal amplitude cutoff. One hundred randomly selected events for each cell (Pyr dTom– and dTom+) were used to create cumulative probability histograms.

### Image analysis

#### Bassoon alignment

PV-Cre x Ai3 mouse brain tissue sections containing FAPpost-transduced cells were stained for bassoon, a presynaptic marker of vesicle-release active zones that localizes to both excitatory and inhibitory synapses (Richter et al., 1999). Images of the four different fluorescence channels (bassoon, PV/YFP, dTom, FAPpost) were arranged side-by-side in series for all optical sections containing the target soma. Analysis was restricted to the surface of a tissue section (∼5 μm from top) where bassoon antibody penetration was complete. In deeper regions of the tissue section, bassoon immunofluorescence was low to undetectable, making colocalization assessments unreliable.

First, bassoon puncta adjacent to the surface of a target dTom-expressing soma were identified by an experimenter. Bassoon puncta sometimes extended across multiple optical sections. Bassoon colocalization at PV/YFP neurites was assessed by direct overlap of signal from the two channels. FAPpost puncta at the soma surface were counted as being associated with bassoon when puncta were aligned with <0.25 μm distance. A minimum of 15 bassoon puncta was assessed for colocalization/alignment for each soma analyzed. We independently examined the rate of FAPpost alignment with bassoon (to identify putative false positives) and PV, as well as PV alignment with bassoon and FAPpost in the same manner.

To assess dendritic FAPpost alignment with synaptic immunofluorescence, spiny dendritic segments running parallel to imaging plane (≥10 μm in length) were manually assessed for alignment between FAPpost and bassoon across one to five flattened confocal sections. Most FAPpost puncta exhibited overlap with bassoon immunofluorescence within this sub-volume. Some FAPpost puncta without an apparent bassoon partner could extend beyond the thin volume assessed. In this minority of cases, additional adjacent optical sections at these specific locations were examined for bassoon signal to determine whether these FAPpost puncta were actual false positives.

#### Imaris segmentation

Carl Zeiss image files were imported into Imaris version 8.4 equipped with the Filament Tracer plugin (Bitplane). The dTom cell fill was used to create a 3D cell-surface rendering using a combination of surface and filament objects. FAPpost puncta were first reconstructed as 3D structures using “surface objects” (to outline puncta borders) created using an estimated 0.5 μm diameter. Due to imaging limitations, only puncta larger than three voxels (∼0.024 μm^3^) were counted, potentially undercounting very small synapses below this detection threshold. Large puncta that potentially reflected smaller, adjacent synapses were separated into multiple objects using the “split touching objects” function with the same estimated 0.5-μm diameter. Thus, large puncta were potentially separated into multiple smaller synapses, a process that could increase the absolute number of detected synapses. Indeed, it is unclear for larger synapses whether these should be counted as a single synapse with multiple active sites and postsynaptic specializations (Tang et al., 2016), or combined into one large synapse (such as the giant synapses observed at the calyx of Held). Puncta were digitally associated with the plasma membrane if their edges lay within 0.5 μm from the soma surface or <1 μm for spiny dendritic regions. Puncta 0.5 μm below cell surface were attributed as cytosolic fluorescence and not included for analysis. Puncta “objects” were then converted into puncta “spots” (with automatic intensity max spot detection thresholds and a 0.5 μm estimated diameter) using surface object centroids in Imaris.

#### Puncta quantification

Puncta densities were quantified for different branch orders. Pyr neurons had only one apical branch segment that was then divided into higher-order branches. The number and length of 2° and higher-order branches analyzed could vary across cells, depending on cell anatomy and image acquisition. For dendritic puncta density averages, values for the Pyr 1° apical dendrite were not included, because this compartment showed a significantly higher density and appeared to be a distinct compartment of the neuron that may be contiguous with the somatic compartment.

#### Automated input assignment

Presynaptic neurite reconstructions were created using automatic background subtraction thresholding of the presynaptic (PV, SST, or VIP) YFP channel, a split-touching objects diameter threshold of 1 μm, and 6 voxel minimum area settings in Imaris. For confocal stacks where presynaptic neurite YFP signal exhibited *z*-axis related signal drop-off, neurite reconstructions using automatic settings were generated separately for superficial and deeper optical sections of the stack. In such cases, both sets of presynaptic neurite reconstructions were visually examined for comparable density and size profiles.

Puncta spots were assigned to a specific presynaptic input using a distance threshold of 0.15 μm from spot centroid to the presynaptic neurite 3D-reconstructions edge. Methods using presynaptic and postsynaptic neurite colocalization may be confounded by false positives (where neurites are near the soma but do not synapse onto it) and false negatives (where a given neurite is associated with two or more postsynaptic sites that are conflated into a single crossing point). Indeed, in many cases, neurites made extended contacts with the soma surface that might include a single or multiple postsynaptic sites. We found that using postsynaptic puncta to differentiate multiple synapses along a large presynaptic neurite enabled a more accurate estimate for the number of putative synaptic contacts.

Because the distance parameters used to identify convergent signals could be digitally adjusted, we explored this space to establish a maximum distance for input detection of 0.15 μm. This was below the diffraction limit for our confocal images. As expected, use of larger distance thresholds for detecting inputs resulted in a substantial increase in the number of assigned puncta. These values were out of range for other published values for inhibitory synapse density and provided confirmation that smaller distance thresholds were more stringent and likely to be more accurate.

#### Statistical analysis

All reported values are mean ± SEM, unless otherwise stated. Dendritic puncta density is mean spot density per linear dendritic segment for a given cell. Soma density is total somatic spot count divided by soma surface area. Density distributions were tested for normality both within and across cells using the Shapiro–Wilk normality test. Within cells, all but two Pyr cells had normally distributed dendritic puncta densities. For these two cells, median dendritic puncta density was used to represent these cell’s average dendritic puncta densities. Mean dendritic puncta density was used for all other cells. One-way repeated measures (RM) ANOVA was used to detect dendritic segment-level dependent puncta density (*p* < 0.05). Pearson’s correlation was employed to test the relationship between Pyr soma surface area and synapse density. Two-way RM ANOVA was used to detect differences in the proportion of input-assigned synapses across Pyr compartments and input-types (*p* < 0.05). *Post hoc* Tukey’s multiple comparison testing was performed to identify significant group mean differences for anatomic data. For physiologic data, unpaired Student’s *t* test was used to identify significant differences in mean mEPSC and mIPSC amplitude and frequency, and Kolmogorov–Smirnov test was used to test for differences in amplitude distributions (*p* < 0.05). All analyses were performed using Origin 2017 statistical software (OriginLab).

## Results

### FAPpost targeting to postsynaptic sites

**Figure 1.**
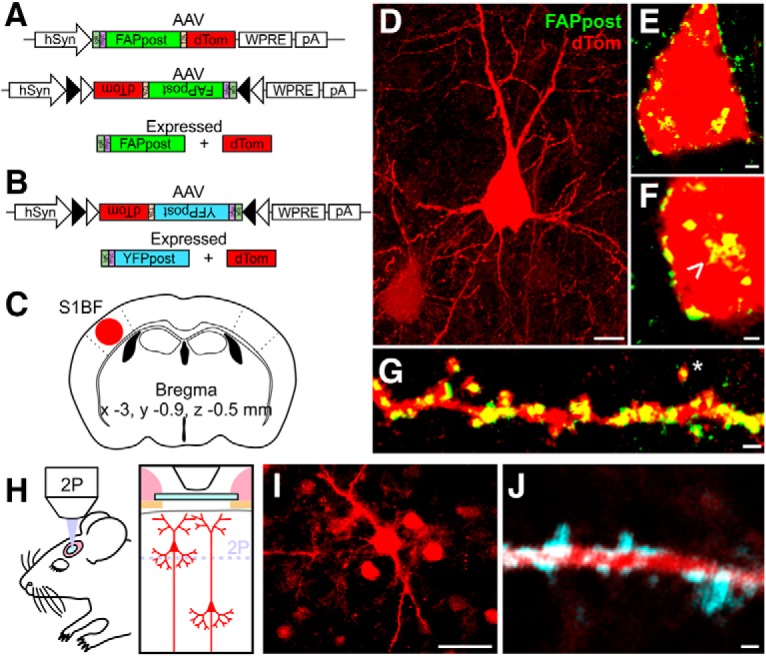
Construct design and expression in mouse somatosensory (S1BF) cortex. ***A***, FAPpost construct design. Human synapsin promotor (hSyn) driving either a constitutively-expressed or Cre-dependent (Fl) FAPpost and dTom, separated by a 2A sequence for independent localization. ***B***, Fl-YFPpost construct. Cre-dependent YFPpost and dTom expression. ***C***, Virus injection coordinates. ***D***, Confocal image stack of L2/3 Pyr cell transfected with FAPpost. Scale bar = 10 μm. ***E***, Optical section of FAPpost puncta on soma of cell in ***D***. Scale bar = 2 μm. ***F***, Zoom of ***E***. Arrowhead marks cytoplasmic FAPpost accumulation. Scale bar = 1 μm. ***G***, FAPpost-labeled spiny dendrites. Scale bar = 1 μm. ***H***, *In vivo* 2P imaging schematic. ***I***, Single-plane 2P image of L2 Pyr cells transfected with of YFPpost and dTom. Scale bar = 30 μm. ***J***, Single-plane 2P image of YFPpost-labeled spiny dendrite in L1. Scale bar as in ***G***. See also Movie 1.

Neuroligins are ubiquitously expressed at postsynaptic sites (Bemben et al., 2015). We took advantage of the pan-synaptic localization of a previously characterized NL-1-based tether (post-mGRASP; (Kim et al., 2011; Druckmann et al., 2014; Kwon et al., 2018) to direct an extracellular fluorophore to postsynaptic sites. Because the trans-synaptic protein-protein interactions involved in GRASP and other protein complementation methods are irreversible and may be linked to synaptic stabilization (Scheiffele et al., 2000; Tsetsenis et al., 2014), we replaced post-mGRASP’s extracellular GFP fragment with an intact FAP or YFP and packaged the modified construct into recombinant AAV virus for expression under the control of the human synapsin promoter (Fig. 1*A*,*B*). Virus was injected into mouse primary somatosensory (barrel) cortex for sparse neuronal labeling (Fig. 1*C*). Transduced cells were identified in fixed tissue specimens using both dTom and FAP expression after MG dye labeling, without further signal amplification (Fig. 1*D–G*; Movie 1).

Movie 1. L2/3 Pyr neuron labeled with dTom and FAPpost. Confocal image stack of an isolated L2/3 neuron with Pyr morphology showing punctate FAPpost (pseudocolored green) along the soma and dendritic arbor. 10.1523/ENEURO.0193-19.2019.video.1

*In vivo* 2P imaging of YFPpost-transduced dendrites from L2/3 Pyr neurons in mouse S1 revealed that punctate YFP signal was associated with both dendritic shafts and spines (Fig. 1*H–J*). Because FAPpost fluorescence required the addition of MG fluorogen, and *in vivo* imaging was conducted using a cranial window with a glass coverslip after several days of recovery, it was not straightforward to image FAPpost expression *in vivo*. Our results indicate that YFPpost is bright enough for detection of puncta in living tissue. Overall, we find that NL-1 tethered fluorophores can be detected in both fixed and living brain tissue without signal amplification, with punctate expression that localizes to sites of synaptic input.

### Synapse localization without functional disruption

Overexpression of other genetically-encoded synaptic proteins has been associated with elevated synapse density and abnormal electrophysiological properties. For example, increased anatomic synapse density and mEPSC frequency has been observed with overexpression of GFP-tagged PSD-95, gephyrin, or intact NL-1 (El-Husseini et al., 2000; Chubykin et al., 2005; Prange et al., 2004; Gross et al., 2013). Trans-synaptic interactions for split protein indicators have been shown to increase binding affinities for the tagged proteins, and irreversible GFP reconstitution can perturb synapse stability and organization (Yamagata and Sanes, 2012; Tsetsenis et al., 2014).

**Figure 2.**
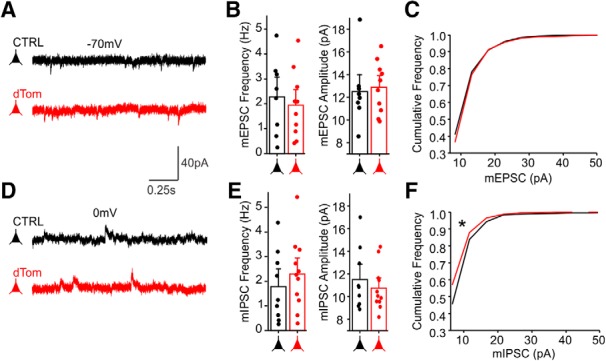
FAPpost synaptic localization does not alter mEPSC and mIPSC properties. ***A***, Example voltage-clamp traces from an untransfected (black) and neighboring FAPpost-expressing (red) L2/3 Pyr cell showing mEPSCs. ***B***, Comparison of mean mEPSC frequency (ANOVA_Frequency_: *F*_(1,16)_ = 0.2, *p* = 0.6) and amplitude (ANOVA_Amplitude_: *F*_(1,16)_ = 0.1, *p* = 0.8) indicate no difference. ***C***, Cumulative distribution histogram of mEPSC amplitudes (Kolmogorov–Smirnov test, D = 0.05, *p* = 0.16). ***D***, Example voltage-clamp traces from an untransfected (black) and neighboring FAPpost-expressing (red) L2/3 Pyr cell showing mIPSCs. ***E***, Mean mIPSCs frequency (left) of untransfected and dTom cells were not significantly different (ANOVA_Frequency_: *F*_(1,18)_ = 0.6, *p* = 0.4). Mean mIPSC amplitude (right) of untransfected and dTom cells were not significantly different (ANOVA_Amplitude_: *F*_(1,18)_ = 0.5, *p* = 0.5). ***F***, Cumulative distribution histogram of mIPSC amplitudes shows a small but significant shift in dTom mIPSC amplitudes (Kolmogorov–Smirnov test, D = 0.15, *p < 0.0001); *n* = 8–11 cells, *N* = 7 animals.

Electrophysiological recordings can be a sensitive way to survey alterations in synaptic function, independent of anatomic quantitation from fluorescence images. To test whether FAPpost expression was associated with altered mEPSC and mIPSC properties, adjacent untransfected and dTom+, FAPpost-transfected cells were targeted for whole-cell recordings (Fig. 2).

FAPpost expression did not alter mean mEPSC frequency or amplitude (Fig. 2*B*; frequency untransfected 2.3 ± 0.5 Hz vs FAPpost 1.9 ± 0.4 Hz; amplitude untransfected 12.5 ± 1.0 pA vs FAPpost 12.8 ± 0.7 pA). Furthermore, mean mIPSC frequency and amplitude were not significantly different (Fig. 2*E*; frequency untransfected 1.8 ± 0.5 Hz vs FAPpost 2.3 ± 0.4 Hz; amplitude untransfected 11.5 ± 0.9 pA vs FAPpost 10.8 ± 0.6 pA), although a small reduction in the frequency distribution of mIPSCs was observed (Fig. 2*F*). Thus, expression of postsynaptic fluorophores using NL-1 targeting sequences can be a non-invasive way to identify and quantitate synaptic distributions without altering synaptic function.

**Figure 3.**
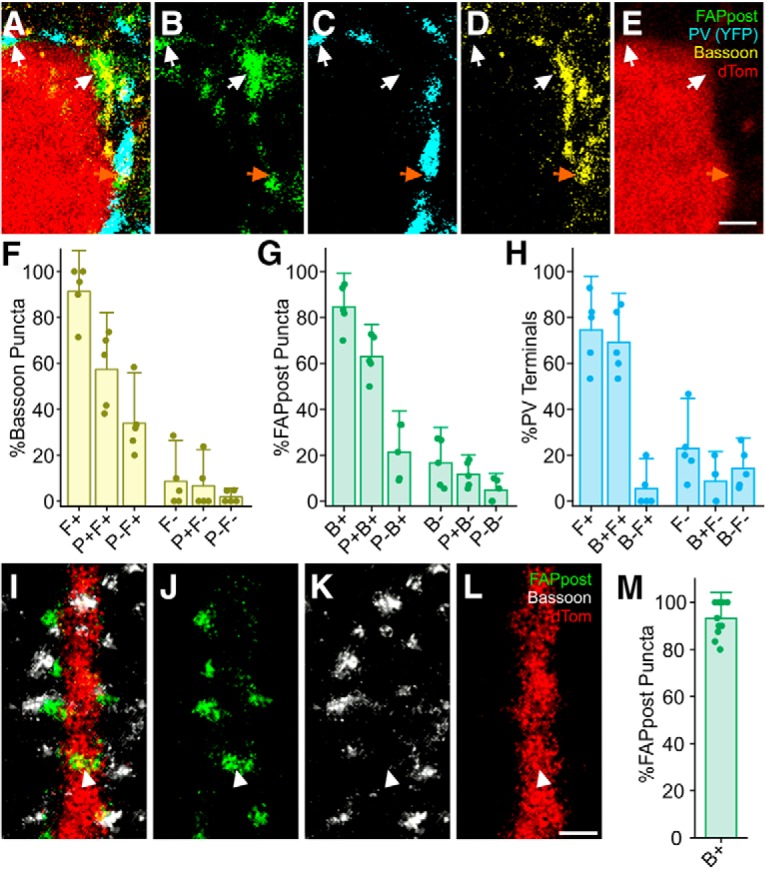
FAPpost puncta align with presynaptic bassoon. ***A***, Optical section of a confocal image used to assess bassoon immunofluorescence alignment with PV terminals on soma of FAPpost-labeled cell visualized in Ai3xPV-Cre mouse. Quadruple channel overlay showing presynaptic PV terminal (YFP, cyan) colocalization with bassoon immunofluorescence (Alexa Fluor 405; yellow) and alignment with FAPpost puncta (green) on dTom (red) filled soma. Scale bar = 1 μm. ***B–E***, As in ***A***, but each channel in isolation. White arrows indicate triple-channel alignment example puncta, orange arrow indicates quadruple-channel alignment example puncta. ***F***, Presynaptic bassoon puncta rate of alignment with FAPpost (F+) and/or colocalization with PV terminals (P+). Bars are mean ± SD of individual soma alignment rates (dots; *n* = 5 soma; puncta assessed, *n* = 104). ***G***, FAPpost puncta rate of alignment with bassoon (B+) and/or PV terminals (P+; dots; *n* = 5 soma; puncta assessed, *n* = 92). ***H***, PV terminal rate of alignment with FAPpost (F+) and colocalization with bassoon (B+, dots; *n* = 5 soma, terminals assessed, *n* = 83). ***I***, Triple channel overlay showing presynaptic bassoon immunofluorescence (Alexa Fluor 405; white) alignment with dendritic FAPpost. Scale bar = 1 μm. ***J–L***, As in ***I***, but each channel in isolation. White arrowhead indicates FAPpost puncta not aligned with bassoon. ***M***, FAPpost rate of alignment with bassoon (B+) along separate dendritic segments (dots; *n* = 11 dendritic segments; puncta assessed, *n* = 143).

To test whether FAPpost synaptic labeling was detecting inhibitory synaptic contacts onto a cell, we evaluated the alignment of FAPpost signal with immunohistochemical detection of a ubiquitous presynaptic marker, bassoon (Richter et al., 1999). We focused on FAPpost labeling at the soma, since synapses here were easily detected in confocal cross-sections. Bassoon immunohistochemistry was conducted in PV-Cre x Ai3 tissue (where PV neurites were labeled with YFP), and bassoon alignment to FAPpost-expressing Pyr neurons in L2/3 was assessed (Fig. 3*A–E*). More than 90% of bassoon puncta aligned with FAPpost puncta (Fig. 3*F*), where only 7 ± 10% (mean ± SD) of bassoon+ PV terminals lacked FAPpost signal. In 3/5 cells analyzed, we observed that all PV terminals showed both bassoon and FAPpost. This suggests that FAPpost labels the vast majority of synaptic contacts from PV neurons. FAPpost puncta showed a slightly lower rate of alignment with presynaptic bassoon (84 ± 10% of detected FAPpost puncta could be aligned to a bassoon puncta; Fig. 3*G*). For FAPpost puncta that did not show bassoon labeling, 12 ± 6% were aligned to PV terminals and were thus likely to be bona fide synaptic contacts. We attribute FAPpost and PV-aligned but bassoon immunonegative terminals to either true bassoon-negative release sites (Dondzillo et al., 2010) or incomplete labeling with the bassoon antibody, either from poor antibody penetration or due to small bassoon puncta that were not detectable given our labeling and imaging conditions. More than 70% of identified PV terminals also showed FAPpost expression (Fig. 3*H*). It is likely that some putative PV terminals apposed to the soma were not actual release sites, since the overwhelming majority (95%) of PV+ terminals aligned with FAPpost also showed presynaptic bassoon signal.

Since our analysis focused on PV inputs at the soma, these data provide evidence that FAPpost effectively labels at least one class of inhibitory synapses. Because we did not evaluate the presence of FAPpost at all synapse types defined by distinct presynaptic and postsynaptic cell types, we cannot be assured that it is equally distributed for all potential synaptic contacts.

We also conducted bassoon immuno-colocalization for FAPpost puncta along spiny dendrites from putative Pyr neurons. Analysis of 11 dendritic segments showed that >90% of FAPpost puncta were associated with bassoon (Fig. 3*I–M*). Because synapses are densely distributed across a volume of brain tissue, and there are many synapses near a labeled segment that belong to unlabeled neurons, it was not possible to determine false negative rates (i.e., bassoon+ but not FAPpost+ for a given dendritic segment). Some FAPpost puncta had no detectable bassoon associated signal. This may result from either an inability to detect bassoon (low fluorescence signal or subthreshold levels of bassoon) or from a bona fide false positive, perhaps due to FAPpost signal that may not be synaptically localized.

### High-throughput synapse quantitation

**Figure 4.**
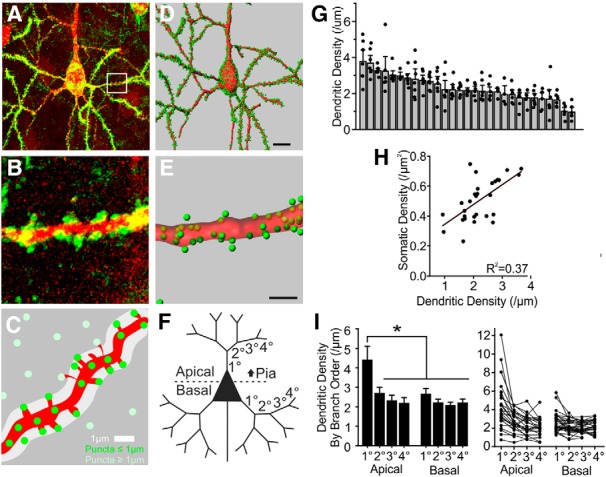
Synapse quantitation for L2/3 Pyr neurons. ***A***, Confocal stack of example FAPpost labeled Pyr neuron. ***B***, Zoom of spiny dendritic segment. ***C***, Schematic for dendritic puncta assignment (green, assigned puncta ≤1.0 μm from shaft surface; light green, unassigned puncta). ***D***, ***E***, 3D rendering of the neuron and puncta assignment. Scale bars = 10 μm, 2 μm. ***F***, Schematic of Pyr branch orders analyzed. ***G***, Mean FAPpost puncta density for individual neurons (gray bars, ±SEM) on 2°–4° apical and 1°–4° basal dendritic branches (black dots). ***H***, Somatic and dendritic puncta density are correlated (*R* ^2^ = 0.37, *p* = 0.0003). ***I***, Mean FAPpost synapse density across 1°–4° Pyr branches (bar ± SEM). Right, Individual cell values, plotted as connected lines. RM ANOVA_Pyr_: *F*_(7,126)_ = 19, *p < 0.0001. All data shown (*n* = 29 cells, *N* = 12 animals), statistical comparisons performed on balanced data (*n* = 19 cells, *N* = 10 animals). See Extended Data Figure 4-1 for puncta detection using YFPpost and Extended Data Figure 4-2 for Imaris analysis workflow.

To facilitate high-throughput quantitative fluorescence analysis of synapses in neurons from brain tissue, we applied an efficient and scalable analysis pipeline with automated synapse detection and assignment. Sparse viral transduction of FAPpost in Pyr neurons from primary somatosensory (barrel) cortex revealed Pyr neurons decorated with bright, FAP puncta across the cell surface (Fig. 4*A*). Using Imaris image analysis software, neural surfaces were rendered and puncta assigned to an individual neuron for quantitative analysis. Because dendritic spines were not always visible from the dTom fill, FAPpost puncta that were 1 μm from the parent dendrite were digitally assigned to a given cell. This semi-automated approach enables high-throughput synapse identification and quantitative analysis (Extended Data Figs. 4-1, 4-2; Fig. 4*D–G*).

10.1523/ENEURO.0193-19.2019.f4-1 Extended Data Figure 4-1 YFPpost fluorescent puncta quantitation in L2/3 Pyr cell dendrites. ***A***, Sparse YFPpost expression across the cortical column. Scale bar = 50 μm. ***B***, L2/3 Pyr-expressing YFPpost. ***C***, YFPpost fluorescent puncta on a dendritic shaft and spines (zoom from box in ***B***). ***D***, Schematic for dendritic puncta assignment (blue, assigned puncta ≤1.0 μm from shaft surface; light blue, unassigned puncta). ***E***, 3D rendering of Pyr neuron (red) with assigned puncta. Scale bar = 20 μm. ***F***, Dendrite from ***C*** with assigned puncta. Scale bar = 2 μm. ***G***, Mean YFPpost puncta density for individual neurons (grey bars, ±SEM) on apical and basal dendritic branches (black dots); *n* = 21 cells, *N* = 4 animals. See also Extended Data Figure 4-2 and Table 1. Download Figure 4-1, TIF file.

10.1523/ENEURO.0193-19.2019.f4-2 Extended Data Figure 4-2 Fluorescent puncta detection method using Imaris. ***A***, Image files imported into Imaris. Scale bar = 40 μm. ***B***, Raw synaptic fluorescent signal. ***C***, Gain-adjusted view of synaptic fluorescent signal to visualize dim YFP signal. ***D***, White mask of synaptic fluorescence differentiates signal from background. ***E***, Puncta creation parameters: 0.5 μm estimated diameter, larger than three voxel size (grey pixels). ***F***, 3D renderings of all fluorescent puncta (yellow). ***G***, Puncta within 0.5 μm from cell surface (edge-to-edge; blue). ***H***, Isolated cell-associated puncta (blue). ***I***, ***J***, Cytoplamic puncta (≤0.5 μm from cell surface; yellow). Scale bar = 5 μm. ***K***, ***L***, Isolated cell-surface puncta. ***M***, Raw synaptic fluorescence used for Imaris spot detection. ***N***, Local fluorescence intensity maxima identified using automatic detection parameters (yellow spots). ***O***, 3D rendering of puncta centers (from ***L***) as spots. ***P***, ***Q***, Alignment of puncta 3D renderings and spots. Scale bar = 1 μm. ***R***, Alignment of spots and signal enhanced fluorescence. ***S***, ***T***, Alignment of spots and raw fluorescence. Scale bar = 5 μm. ***U***, Workflow summary. Download Figure 4-2, TIF file.

Reconstructions of fixed specimens yielded 200–1000 μm of continuous dendritic segment per neuron for analysis. Overall, FAPpost puncta densities across L2/3 Pyr dendrites, 2.3 ± 0.1 puncta/μm (excluding the 1° apical dendrite; see Materials and Methods) were similar to previous estimates of synapse density (Hersch and White, 1981; Gulyás et al., 1999; Holtmaat et al., 2005; Kasthuri et al., 2015; Villa et al., 2016), supporting this high-throughput analytical approach. We also examined puncta densities in L2/3 Pyr neurons from the Emx1-Cre transgenic mouse strain using a Cre-dependent YFPpost construct (Extended Data Fig. 4-2). On average, puncta densities were elevated for L2/3 Pyr neurons in this strain (2.9 ± 0.1 puncta/μm), consistent with the elevated spontaneous activity and enhanced seizure susceptibility that has been observed in this strain (Kim et al., 2013; Steinmetz et al., 2017).

We observed substantial heterogeneity in detected puncta density across individual L2/3 Pyr neurons, with close to four-fold variance across cells (1.0–3.8 puncta/μm), and nearly 10-fold variance (0.7–5.9 puncta/μm) across different dendritic branches (Fig. 4*G*). We found no relationship between mean puncta density and total dendritic length analyzed for a given neuron. Variability in overall puncta density for any cell type could not generally be explained by sex, age, days postinfection, or animal-to-animal differences as neurons with a range of puncta densities could be found in the same animal (Table 1). It is likely that the number of viral particles infecting individual neurons was not uniform, even in cells analyzed from the same animal, and may be an additional source of heterogeneity in analysis of puncta densities; however, we found no significant relationship between puncta intensity and puncta density across analyzed neurons. Dendritic puncta density for a given cell was correlated with the soma density (*R* ^2^ = 0.37). Variability in observed puncta density for Pyr neurons is consistent with anatomic and electrophysiological response variability that has been described for this group of (Yassin et al., 2010; Yamashita et al., 2013; Prönneke et al., 2015; Tyler et al., 2015; van Aerde and Feldmeyer, 2015; Tasic et al., 2016), and may reflect both developmental and molecular heterogeneity of neocortical Pyr neurons.

Although we did not systematically evaluate FAPpost properties in inhibitory neurons, we observed punctate fluorescence in PV, SST, and VIP neurons that expressed this postsynaptic label. Thus, this tool may be useful for quantitative synapse analysis in multiple cell types.

Quantitative analysis across different dendritic compartments revealed that the primary (1°) apical dendrite, a short region of dendrite that emerges from the soma of Pyr neurons, showed dense FAPpost puncta (Fig. 4*I*). The high density of putative synapses in this compartment has not been well-described, in part because prior analyses have typically used dendritic spines as a proxy for synapses and this region is characteristically smooth. Mean puncta density on higher-order dendritic branches was similar across segments.

### Presynaptic input assignment using fluorescence-based colocalization

Transsynaptic molecular complementation for synapse detection requires both presynaptic and postsynaptic transgene expression and may introduce unwanted effects on synapse function. In addition, because presynaptic input labels are not typically saturated using virally-introduced transgenes, complete and quantitative comparisons of input densities across cells and conditions are difficult. Here we assessed whether dendritic FAPpost could be used for identification of putative synaptic contacts where presynaptic neurites are fully labeled using Cre-dependent YFP expression in transgenic mice (Hippenmeyer et al., 2005; Taniguchi et al., 2011). The far-red emission of MG-binding FAPs can be easily multiplexed with other commonly-used fluorophores for detection of adjacent presynaptic and postsynaptic signals. Although many other studies have used the convergence of presynaptic and postsynaptic histochemical or fluorescence signal (Schoonover et al., 2014; Kubota et al., 2015) the introduction of a third feature that marks putative synapse location should improve the accuracy of input-specific synapse assignment in genetically-selectable, sparsely-labeled target cells.

**Figure 5.**
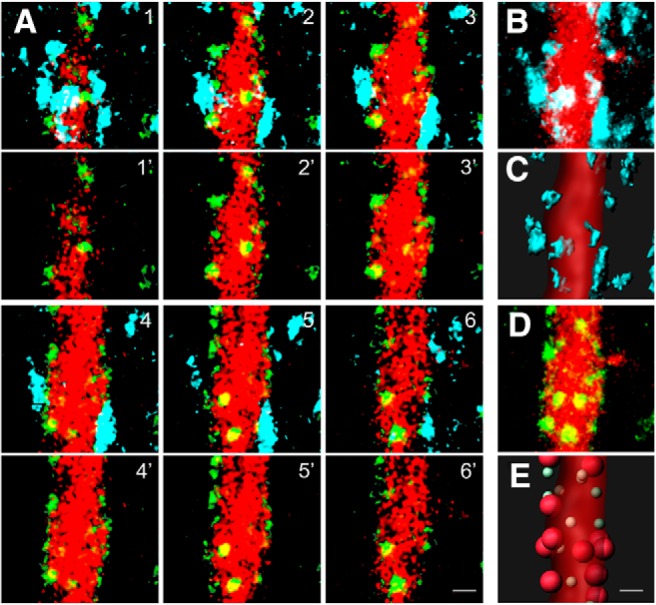
FAPpost puncta on the primary apical dendrite align with presynaptic PV neurites. ***A***, Six serial optical sections of a Pyr primary apical dendrite labeled with FAPpost (green) and dTom (red). Top row, Fluorescence aligned with presynaptic PV (YFP; cyan). Bottom row, FAPpost and dTom fluorescence alone. ***B***, Flattened stack of the region in ***A***, showing PV(YFP) and dTom. ***C***, Rendering of ***B***. ***D***, As in ***B***, but for FAPpost and dTom. ***E***, Rendering of PV-assigned FAPpost puncta (large red balls) and unassigned (small green balls) puncta. Scale bar = 1 μm.

Initially, we focused on the primary apical dendrite of L2/3 Pyr neurons, where FAPpost puncta were clearly demarcated and densely distributed. Manual inspection of a confocal image series shows YFP-labeled PV neurites associated with the dendrite in close proximity to FAPpost puncta (Fig. 5*A*). Digital analysis of this 3D segment for both puncta detection and neurite surface rendering enables a distance-based criterion for assigning specific puncta to PV inputs (Fig. 5*B–E*). A limitation of this fluorescence-based approach, for both us and in previous studies, is that diffraction–limited images cannot perfectly differentiate between apparent and true synaptic contacts. However, use of consistent analysis parameters across specimens may be sufficient to detect condition-specific changes in input organization.

How does inclusion of synaptic markers improve the detection of putative synapses? We compared the number of detected contacts using only fluorescence labeling of presynaptic neurites and the postsynaptic cell, or using these two features plus the presence of a FAPpost puncta at a contact site. We hypothesized that the number of putative synaptic contacts would be reduced when a third feature was required for synapse detection, and thus this method might offer improvements on prior quantitative approaches. Analysis focused on YFP-labeled inputs to Pyr soma, where data from prior EM and light microscopy analyses could confirm our analysis (Tamás et al., 2000; Di Cristo et al., 2004; Melchitzky and Lewis, 2008; Hill et al., 2012; Kubota et al., 2015, 2016; Zhou et al., 2017).

**Figure 6.**
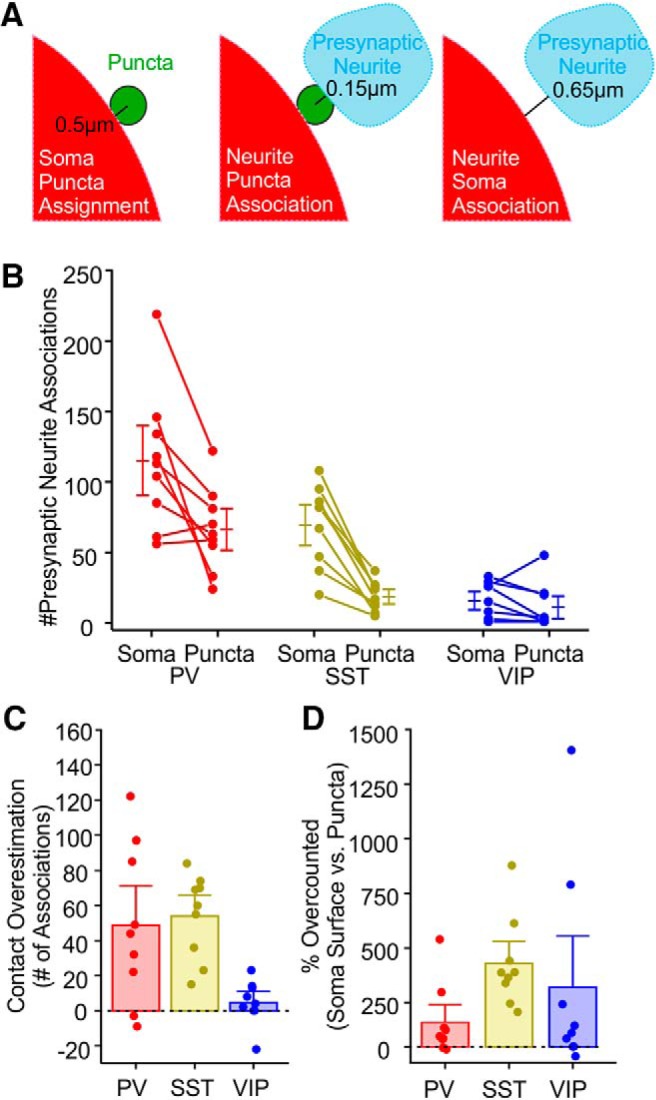
FAPpost detection improves estimates of input association. ***A***, Diagram illustrating distance parameters used for FAPpost puncta assignment to soma-surface (left), soma-puncta association with presynaptic YFP-expressing neurites (middle), and presynaptic neurite associations with soma-surface (right). ***B***, Comparison of the number of presynaptic neurite to soma-surface and soma-puncta associations. Connected lines = individual cell values. More PV neurite to PYR soma-surface (115 ± 16) than soma-puncta contacts were detected (66 ± 30; paired *t* test, *t* = 3.3, *p* = 0.01). More SST neurite to soma-surface (69 ± 10) than soma-puncta contacts were detected (15 ± 8; paired *t* test, *t* = 6.7, *p* = 0.0001). Number of VIP neurite to soma-surface (16 ± 4) and soma-puncta contacts were similar (11 ± 5; paired *t* test, *t* = 1.1, *p* = 0.3). ***C***, Contact overestimation depicts the difference between presynaptic neurite to soma-surface and soma-puncta associations for presynaptic PV neurites (+49 ± 15), SST neurites (+54 ± 8), and VIP neurites (+5 ± 4). Negative values occurred when multiple puncta were associated with a single presynaptic terminal. ***D***, Error rate for presynaptic neurite-to-soma associations versus soma puncta associations for PV (115 ± 56%), SST (394 ± 70%), and VIP inputs (280 ± 162%). Across PYR cells, the percentage overestimation did not vary by input cell type (ANOVA_CellType_: *F*_(2,24)_ = 1.7, *p* = 0.2). PV input: *n* = 9 cells, *N* = 4 animals; SST input: *n* = 9 cells, *N* = 4 animals; and VIP input: *n* = 9 cells, *N* = 3 animals.

Using only neurite-associations with the postsynaptic soma, a method that has been frequently used to estimate PV cell innervation (Di Cristo et al., 2004; Feldmeyer et al., 2006; Hill et al., 2012), we estimated the number of putative somatic synapses for individual Pyr neurons. We then compared these values for the same cell with additional requirement of a FAPpost puncta in between the neurite and the postsynaptic soma, using a neurite-to-puncta distance detection threshold of 0.15 μm (Fig. 6). The use of three features (presynaptic neurite, postsynaptic puncta, and postsynaptic neuron) to quantify input density reduced the number of putative contacts, or false positives, that likely result from non-synaptic neurite juxtaposition. In addition, we sometimes observed neurite apposition at the soma that was associated with multiple underlying FAPpost puncta, suggesting that prior methods using only presynaptic and postsynaptic proximity might have underestimated actual synapse number. Overall, this analysis enabled us to obtain quantitative information about synapse number using fluorescence imaging data more accurately than would be provided by only presynaptic and postsynaptic neurite apposition.

We used this quantitative data to compare the density of inhibitory inputs to the soma across three classes of inhibitory neurons. FAPpost puncta at the soma were >4-fold more likely to be aligned with PV than SST neurites (mean ± SD, somatic PV-assigned puncta 66 ± 30, *n* = 9 Pyr neurons; vs somatic SST-assigned puncta 15 ± 8, *n* = 9 Pyr neurons; Fig. 6). Analysis of VIP-associated inputs revealed a small number of colocalized postsynaptic puncta at the soma (somatic VIP-assigned puncta 11 ± 16, <5% of total somatic puncta; *n* = 9 Pyr neurons). Taken together, approximately one-third of somatic puncta could be assigned to either PV, SST, or VIP inputs; the stringency of our input-detection parameters likely underestimates the number of contacts, particularly for PV neurons. Our findings are consistent with prior reports showing that the majority of somatic inputs arise from PV neurons, with a minority of other inhibitory inputs (Micheva and Beaulieu, 1995; Di Cristo et al., 2004; Melchitzky and Lewis, 2008; Hill et al., 2012; Kubota et al., 2016), and show that synapse identification improves the accuracy of quantitative input analysis.

### Somatic and dendritic inhibition is dominated by PV input

**Figure 7.**
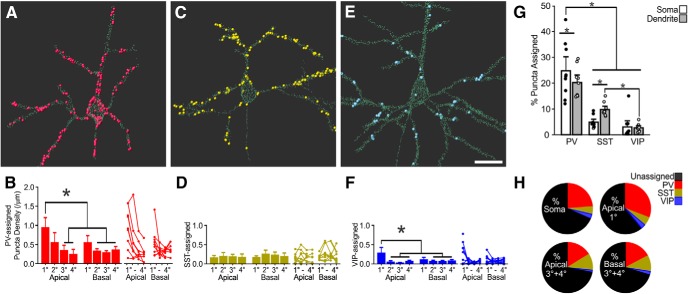
The distribution of PV, SST, and VIP inputs across L2/3 Pyr neurons. ***A***, PV-input assigned synapses for an example L2/3 Pyr neuron. Small light-green spheres are un-assigned FAPpost puncta; large colored spheres are input-assigned FAPpost puncta. See Extended Data Figure 7-1 for images of all input-analyzed Pyr neurons. ***B***, Mean density of PV-assigned FAPpost contacts across dendritic branch orders. Left, Bar is mean ± SEM of all cells. Right, Individual cell values, plotted as connected lines. All data shown, statistical comparisons performed on balanced data. PV-assigned puncta density was greater for the 1° apical dendrite. RM ANOVA_PV-Input_: *F*_(7,28)_ = 6.7, *p* = 0.002; *n* = 5 cells, *N* = 3 animals. *, Tukey *post hoc* pairwise comparison test, *p* < 0.05. ***C***, As in ***A***, but for SST. See Extended Data Figure 7-2 for images of all input-analyzed Pyr neurons. ***D***, SST-assigned puncta density was not statistically significantly different across branch orders. RM ANOVA_SST-Input_: *F*_(7,28)_ = 0.63, *p* = 0.7; *n* = 5 cells, *N* = 3 animals. ***E***, As in ***A***, but for VIP. Scale bar = 20 μm. See Extended Data Figure 7-3 for images of all input-analyzed Pyr neurons. ***F***, VIP-assigned puncta density was greater for the 1° apical dendrite. RM ANOVA_VIP-Input_: *F*_(7,42)_ = 3.8, *p* = 0.003; *n* = 7 cells, *N* = 3 animals. *, Tukey *post hoc* pairwise comparison test, *p* < 0.05. ***G***, Inhibitory innervation of Pyr neurons, expressed as a percentage of the total number of detected synapses, for each input source. All dendritic compartments pooled for somatic and dendritic comparison. Two-way RM ANOVA_Input_: *F*_(2,24)_ = 45, *p* < 0.001. All * show Tukey *post hoc* pairwise comparison test, *p* < 0.05. ***H***, Pie-chart showing average proportion of input-assigned FAPpost contacts versus total detected synapses, binned as perisomatic (soma and 1° apical) or higher-order dendritic compartments (apical 3° + 4° and basal 3° + 4°). A greater proportion of PV-inputs were found on soma and 1° apical dendrite than for SST- or VIP-inputs. At higher-order apical branches, the proportion of SST-input (10.5 ± 1.9%) was similar to PV-input (16.2 ± 1.9%), but significantly greater than VIP-inputs (1.4 ± 1.9%). For higher-order basal branches, all input sources were significantly different (PV = 17.3 ± 1.4%, *n* = 7 cells, *N* = 4 animals; SST = 10.2 ± 1.4%, *n* = 7 cells, *N* = 4 animals; VIP = 2.3 ± 1.4%, *n* = 7 cells, *N* = 2 animals). Two-way RM ANOVA_Binned-Input_: *F*_(2,18)_ = 47.3, *p* < 0.0001.

It is commonly held that PV inputs preferentially target the soma and SST inputs, the dendrites (particularly in L1; Pi et al., 2013; Chen et al., 2015; Dienel and Lewis, 2019). However, quantitative evidence for this is lacking and indeed recent reports suggest that PV inputs may be broadly arrayed across the dendritic arbor (Kubota et al., 2015). We compared the distribution of PV, SST, and VIP inputs across the soma and along Pyr dendrites, including 4° branches that could extend >140 μm from the soma center (Fig. 7, with gallery of PV-assigned, SST-assigned, and VIP-assigned inputs on individual L2/3 Pyr neurons in Extended Data Figs. 7-1, 7-2, 7-3).

10.1523/ENEURO.0193-19.2019.f7-1 Extended Data Figure 7-1 PV-assigned FAPpost puncta distribution across Pyr cells. Small light-green spheres are un-assigned FAPpost puncta. Large colored spheres are input-assigned FAPpost puncta. Scale bar = 15 μm. Download Figure 7-1, TIF file.

10.1523/ENEURO.0193-19.2019.f7-2 Extended Data Figure 7-2 SST-assigned FAPpost puncta distribution across Pyr cells. Small light-green spheres are un-assigned FAPpost puncta. Large colored spheres are input-assigned FAPpost puncta. Scale bar = 15 μm. Download Figure 7-2, TIF file.

10.1523/ENEURO.0193-19.2019.f7-3 Extended Data Figure 7-3 VIP-assigned FAPpost puncta distribution across Pyr cells. Small light-green spheres are un-assigned FAPpost puncta. Large colored spheres are input-assigned FAPpost puncta. Scale bar = 15 μm. Download Figure 7-3, TIF file.

Because soma size could vary more than two-fold between neurons (complicating measures of density), we compared the percentage of total puncta that could be assigned to PV, SST, or VIP inputs for individual Pyr soma. Somatic puncta were dominated by PV inputs, where on average 25% of FAPpost puncta could be assigned to adjacent PV neurites but only 5% of FAPpost puncta could be assigned to SST inputs. In general, VIP inputs were rarely observed on L2/3 Pyr soma (Fig. 7*C*,*F*).

Quantitative input assignment revealed that PV inputs were frequently observed along all dendrites where their distribution only modestly declined at higher branch orders (up to 4° branches; Fig. 7*F*,*H*). On average, dendritic SST inputs were less abundant than PV inputs, using either density measurements (mean density dendritic SST-assigned puncta 0.20 ± 0.03/μm vs PV-assigned puncta 0.38 ± 0.07/μm, excluding the 1° apical dendrite for both) or relative proportion of assigned puncta (Fig. 7*G*). Even in higher-order (3° and 4°) apical dendrites, SST inputs were not more numerous than PV inputs (Fig. 7*H*). It remains possible that the L1 apical tuft of L2/3 Pyr neurons (that was not included in our analysis, due to sectioning artifacts) may show denser SST inputs. Overall, quantitative input analysis shows that both PV and SST inputs are broadly distributed across the proximal dendrites of Pyr neurons within L2/3.

Interestingly, we observed a pronounced concentration of PV inputs at the synapse-dense 1° apical dendrite as it emerged from the soma in L2/3 Pyr neurons (Fig. 7*A*,*B*,*H*), with a significant 6-fold greater density than for SST inputs. These data suggest that the 1° apical compartment might be an extension of the soma with respect to PV presynaptic targeting and synaptic integration properties. The prominent absence of SST inputs at the somatic and 1° apical dendrite suggests that SST neurons may selectively avoid these PV-input-enriched perisomatic compartments (Fig. 7*G*,*H*). Cortical wiring diagrams showing SST input are frequently schematized to indicate the apical dendrite or L1 as the primary site of synaptic input (Pi et al., 2013; Chen et al., 2015; Dienel and Lewis, 2019). Our data indicate that SST inputs are detectable across the dendritic arbor and may not be restricted to this layer.

VIP inputs to Pyr neurons showed a slightly higher density for the 1° apical versus other dendrites, although the absolute number of synapses was very low. Overall, VIP input density was 10-fold lower than PV inputs to the 1° apical dendrite, a difference that was highly significant (Fig. 7*G*). For higher-order dendrites, VIP input density was significantly lower than either PV and SST inputs (mean density, dendritic VIP-assigned puncta excluding the 1° apical dendrite 0.07 ± 0.01/μm). These differences were also reflected in the relative proportion of assigned FAPpost puncta. For example, at higher-order apical dendrites the proportion of total SST-assigned puncta (10.5 ± 1.9% of total inputs) was slightly lower than the proportion of PV-assigned puncta (16.2 ± 1.9% of total inputs), but significantly greater than VIP inputs (1.4 ± 1.9%).

Because there are a small fraction of PV-Cre expressing Pyr neurons in deep layers, it is possible that some of the detected PV inputs may arise from PV-expressing Pyr neurons. However, PV-expressing Pyr neurons are not observed in L2/3, and it is likely that the majority of PV inputs arise from intralaminar inputs (Fino and Yuste, 2011). These findings are consistent with meticulous neuroanatomical reconstructions of synaptically-connected pairs showing that PV synapses can be observed across the dendritic arbor of neocortical Pyr neurons (Kubota et al., 2015). In addition, our data indicate that SST inputs are common across the dendritic arbor within L2/3. These data suggest revisions to previous cortical wiring diagrams that show SST inputs exclusively at the apical tuft and PV inputs exclusively at the soma (Pi et al., 2013; Chen et al., 2015; Dienel and Lewis, 2019).

## Discussion

Synapses are a critical determinant of neural function, and their individual and collective properties can provide insight into how brain circuits are organized and changed by experience. Electrophysiological measurements of mEPSC and mIPSCs have been widely used to assess circuit-level adaptations in synaptic function, but typically sample only a small subset of inputs onto a neuron close to the recording electrode due to electrical filtering of small and distant signals. In contrast, anatomic methods offer a highly quantitative, compartment-specific and anatomically broad view of how synapses and cell type-specific inputs are distributed onto a neuron.

A fluorescence-based, molecular genetic platform for synaptic detection and quantitation has multiple advantages for high-throughput and scalable analysis. First, the brightness of FAP/YFPpost synaptic tags enable direct visualization of synapses in both live and fixed tissue without amplification, making them accessible tools for broad scale use. Second, synaptically-targeted fluorophores can be sparsely expressed in brain tissue, not just cultured neurons, to reveal properties of synaptic and input organization in a complex neural circuit. Third, fluorescence imaging enables use of multiple, spectrally distinct channels for cell type-selective identification of axonal inputs and specific molecules that can differentiate synapses. Fourth, volumetric data collection is rapid and requires only a confocal microscope, and images can be used for high-throughput, automated analysis. Overall, quantitative and high-throughput synapse detection with FAP/YFPpost will facilitate cell type-specific characterization of synapses and connectivity changes across multiple animals and diverse experimental conditions.

### Synaptic quantitation without perturbation

Experimental evidence indicates that FAPpost labels both excitatory and inhibitory synapses. FAPpost puncta are localized to soma and dendritic shafts, preferred targets for inhibitory synapses as well as on dendritic spines where excitatory synapses lie. FAPpost convergence with presynaptic inputs from confirmed GABAergic neuron subtypes, specifically PV, SST, and VIP neurons, indicates association with inhibitory inputs. The FAPpost labeling of both excitatory and inhibitory synapses allows comprehensive analysis of synapse distribution from a single postsynaptic marker. Reagents that separately enable visualization of excitatory and inhibitory synapses will also be useful tools for fluorescence-based quantitative imaging (Gross et al., 2013; Chen et al., 2015), if expression levels are high enough for reliable synapse detection.

Although it has been proposed that NL-1 is specifically targeted to excitatory synapses (Song et al., 1999), NL-1 contains a conserved binding motif for the GABAergic receptor scaffolding molecule gephyrin and NL-1-based synapse labeling constructs are sometimes observed at inhibitory synapses (Tsetsenis et al., 2014; Bemben et al., 2015; Kwon et al., 2018). Neurexin 1β-binding to the extracellular portion of NL-1 has been shown to enhance intracellular PSD-95 interactions (Giannone et al., 2013) and this region’s deletion in FAPpost may enable the broader distribution observed in transduced neurons. Importantly, prior studies have shown that the absence of the extracellular NL-1 region inhibits ectopic synapse formation (Chih et al., 2005), supporting the use of FAPpost as a non-invasive tag for synapse monitoring.

Does FAPpost expression alter synaptic function *in vivo*? This is a significant issue, as the electrophysiological effects of other fluorescence synapse detection reagents have not been well-investigated (Kim et al., 2011; Martell et al., 2016; Choi et al., 2018). Overexpression of tagged synaptic molecules leads to an increase in overall synapse number, using electrophysiological or anatomic measurements (El-Husseini et al., 2000; Gross et al., 2013). In such cases, quantitative analysis can be misleading, reflecting either a primary overexpression effect or a secondary effect of circuit-level adjustments to abnormal synaptic input. The absence of a clear electrophysiological phenotype for the FAPpost reagent suggests that overexpression of our fluorophore-tagged NL-1, in the absence of trans-synaptic interactions, may have a minimal effect on synaptic function.

### Synapse detection accuracy

How does FAPpost synaptic quantitation compare to previous estimates of synaptic density and input organization? Synapse density has often been estimated indirectly from fluorescence images, using spines as a proxy for synapses. This is problematic, as spine detection will underestimate synapse number by excluding shaft synapses (typically inhibitory), dually-innervated spines (>10% by some estimates (Chen et al., 2012)), spines that lie within the imaging plane, and will also undercount faintly labeled, filamentous spines.

Overall synapse densities for L2/3 Pyr neurons revealed an average of 2.5 synapses/μm dendrite, a density that is well within the range of prior estimates. For example, whole-cell EM reconstructions of CA1 hippocampal neurons have shown synapse densities of 0.7–7 synapses/μm, depending on location within the dendrite (Gulyás et al., 1999). Other studies analyzing spine (not synapse) density from L2/3 or L5 Pyr neurons in mouse S1 report between 0.4 and 5.1 spines/μm, where EM studies typically reveal greater spine densities (Holtmaat et al., 2005; Kasthuri et al., 2015; Villa et al., 2016).

Fluorescence-based synaptic tags reduce multiple sources of error that can inaccurately assess total synapse number. For isolated neurons, automated puncta assignment to the parent dendrite removes the requirement that dendritic spines be visible for synapse detection, reducing false-negative rates. For automated assignment, this rate will always be non-zero as the distance limit set for puncta assignment will exclude puncta that lie on longer dendritic spines, which can extend >5 μm in some cases (Kasthuri et al., 2015).

False-positive (non-synaptic puncta) errors are more difficult to estimate. Intracellular pools of the targeting construct may contribute. Although these were digitally excluded based on distance to the plasma membrane, this process that may not be effective for thin dendritic segments. It is possible that non-synaptic, plasma-membrane FAPpost accumulation may sometimes occur. In addition, puncta from nearby neurons may have been inadvertently misassigned to an analyzed dendritic segment. While fluorescence-based genetic methods have advantages, they are subject to variations in expression levels, both of the labeling construct and of protein trafficking to different synapses (for example, that may have a lower NL-1 content or are more distant from the soma). It remains possible that not all synapses, for example, neuromodulatory or peptidergic inputs, were uniformly labeled using this methodology. The quantitative analysis pipeline established here attempts to reconcile high-throughput analysis with variability in synapse structure, where speed and accuracy must be balanced.

### Biological and non-biological variability

We observed marked within (10–20-fold) and across cell (2–4-fold) variability in FAP/YFPpost synapse density for L2/3 Pyr neurons. Variability in synapse density and its biological implications has not been well-explored. Most analyses have focused on complete reconstruction of a single neuron (Megías et al., 2001) or of a few dendritic segments (Kasthuri et al., 2015; Villa et al., 2016). In one study that conducted a detailed analysis of multiple Pyr cells’ apical dendrites, a wide range of synapse densities were observed (Hersch and White, 1981). Cells with higher synapse densities may represent “hub” cells that receive wide distribution of synaptic inputs, or more recurrent connections from the same presynaptic neuron(s). Notably, miniature postsynaptic current frequency data (both ours and others) shows a 10-fold range in values across Pyr neurons. Mini frequencies are typically interpreted as reflecting the number of synaptic connections on a given postsynaptic cell. Considering the electrophysiological correlate of synapse number shows a similar range in values to anatomic correlates of synapses on Pyr neurons, we may very well be capturing normal biological variability in synapse numbers.

Alternatively, observed variability may be a non-biological labeling artifact. To achieve complete synaptic labeling, expression of any synapse-tagging molecule must reach sufficient levels in an individual cell to label all synapses across its entire dendritic arbor. It is unlikely that this labeling occurs at the same time for all cells. One alternative explanation for the wide-range in synapse densities is that only a fraction of synapses was labeled in a particular cell. We attempted to control for this by only selecting well-labeled cells, but we cannot rule out this potential confound in the interpretation of our findings.

### Volumetric imaging for high-throughput synaptic input assignment

A significant advance enabled by an all-fluorescence synaptic imaging platform is the automated assignment of cell type-specific synaptic inputs with a spectrally distinct fluorophore. The tricolor (FAPpost, dTom, and presynaptic YFP) association as a criterion for synapse detection substantially reduces the false positive rate compared to brightfield microscopy methods (Hill et al., 2012; Schoonover et al., 2014; Kubota et al., 2015). We took advantage of the saturated labeling of molecularly-defined inhibitory neuron populations in Cre-driver transgenic mouse lines to examine the broad-scale distribution of FAPpost-labeled synaptic inputs on L2/3 Pyr neurons that originated from three types of GABAergic neurons.

Our analysis revealed that PV inputs predominate at somatic locations, with more than four times as many PV as SST inputs to the cell body. These data are consistent with reports of dense PV innervation of the soma (Kubota et al., 2016) and a small fraction of somatic SST inputs (<10%; Hill et al., 2012), and further validate FAPpost labeling as a robust method for quantitative synapse assignment. Dendritic analysis of synapse organization identified the 1° apical dendrite of Pyr neurons as a site of particularly dense PV innervation. This aspiny region of the dendrite, particularly in neocortical Pyr neurons, has been poorly studied as prior imaging methods have not been able to reliably visualize synapses in this compartment. Importantly, whole-neuron EM reconstructions show that >90% of inputs to the apical dendrite of CA1 neurons are inhibitory (Megías et al., 2001; Bloss et al., 2016). The distinctive properties of the apical dendrite (Major et al., 2013) suggest that PV input to this region may serve as a critical filter for top-down modulation of Pyr neuron firing in the neocortex.

Quantitative analysis showed that PV neurons have more input to Pyr neuron dendrites than other neocortical inhibitory neurons. Indeed, even when excluding the densely PV-innervated 1° apical dendrite, mean dendritic input density was greater for PV than SST, and VIP inputs. Although this may be incongruous with the simplified model that contrasts soma-targeting PV and dendrite-targeting SST inputs (Lazarus and Huang, 2011; Pi et al., 2013; Higley, 2014; Chen et al., 2015; Pakan et al., 2016), prior experimental data are much less categorical than these schema suggest. For example, anatomic reconstructions from paired whole-cell recordings show that the majority of PV inputs to neocortical Pyr neurons are located >50 μm from the soma (Hill et al., 2012; Kubota et al., 2015) and abundant SST contacts can be detected at both proximal and distal dendrites (Di Cristo et al., 2004; Hill et al., 2012). It remains possible that very distal dendrites, particularly in L1, have a disproportionate association of SST inputs. Based on the density of synaptic inputs, our data indicate that PV-mediated synaptic input will be the predominant source of inhibition across the somatodendritic compartments of L2/3 Pyr neurons.

## Conclusion

This analysis helps generate a framework for large-scale anatomic imaging to examine circuit- and brain-wide changes in synapse distribution in development, learning, and disease. Future efforts should leverage volumetric imaging in cleared or expanded tissue for complete and high-resolution capture of the entire dendritic apparatus, application of additional molecular markers to distinguish different synapse types, and employ new presynaptic constructs for improved synaptic discrimination. A critical challenge of these future possibilities will be the digital capture and storage of large anatomic datasets for computational analysis.

## Synthesis

Reviewing Editor: Alfonso Araque, University of Minnesota Decisions are customarily a result of the Reviewing Editor and the peer reviewers coming together and discussing their recommendations until a consensus is reached. When revisions are invited, a fact-based synthesis statement explaining their decision and outlining what is needed to prepare a revision will be listed below. The following reviewer(s) agreed to reveal their identity: Pavel Osten.

The reviewers have found merit and interest on the findings reported. I concur with the reviewers' comments expressing the interest of the study. However, they also expressed some concerns about several issues that need to be addressed and clarified. I also consider pertinent the concerns expressed.

Specific comments of the reviewers:

Reviewer 1.

In this manuscript, the authors developed a method for labeling and analyzing synaptic connectivity by expressing fluorogen-activating protein (FAP) or YFP linked to postsynaptically-localized neuroligin-1 targeting sequence. They show that overexpressing FAP at postsynaptic sites (FAPpost) has minimum effects on mEPSC or mIPSC in pyramidal neurons. The expression of FAPpost or YFPpost allows better detection of putative synaptic sites based on postsynaptic markers, rather than simply morphological makers. Using an automated detection algorism in Imaris software, the method enables detection of synaptic connections between several types of inhibitory neurons and L2 pyramidal neurons. The authors demonstrate the usefulness of this method by showing that PV neurons make synaptic contacts on apical dendrites in addition to somas of L2 pyramidal neurons, whereas SST-expressing interneurons target both proximal and higher-order dendrites of pyramidal neurons.

The new method developed enables potentially more precise analysis of fluorescently-labeled synaptic connections in the brain. The results are clearly presented and properly discussed. With some further characterization, the method could be very effective for rapid and large scale analysis of synaptic connectivity. I only have few suggestions for improving the manuscript:

(1) The authors show FAPpost expression can be imaged after the addition of MG fluorogen to the fixed tissue but not in the living cortex. It is unclear whether this is purely due to rapid clearance of MG fluorogen in vivo or just the weak labeling intensity for two-photon imaging. Could the authors test whether immediately after adding MG fluorogen, FAPpost could be imaged in vivo with two-photon microscopy?

(2) As the authors acknowledged, the variability of spine density across different cells could be due to the level of FAPpost expression. Presumably, spine density could be independently measured with the cell-filling marker dTomato. If the dTomato expression is weak, the authors may consider using antibody staining to amplify the dTomato signal. If every spine identified by dTomato was also labeled by FAPpost, this would suggest that the variability of spine density is not due to the level of FAPpost expression.

(3) What level of FAPpost expression in cells is appropriate for imaging and data analysis? The authors should mention this in the method or the main text. Does the brightness of FAPpost correlate with spine density detected on pyramidal neurons?

(4) Does FAPpost label synapses in inhibitory neurons? The authors should at least comment on this issue.

Reviewer 2.

In the paper the authors developed a method for fluorescence-synapse and connectivity labeling and analysis using Fluorogen activating protein (FAP)- or YFP-tagged postsynaptic neuroligin-1. They show that this largely does not change mEPSC or mIPSC properties and they use the method to visualize synaptic sites of PV, SST and VIP interneurons onto L2 pyramidal neurons in mouse somatosensory cortex.

This is an overall nice study that could be further improved with a few more experiments on the validation of the observed FAP/YFP postsynaptic labeling. For example, the image in Fig 3A-E shows what appears to be a soma with examples of presynaptic bassoon colocalization for 3 putative synaptic sites, but this is not representative of the distribution on dendrites that is being quantified in the rest of the manuscript. Therefore it would be more appropriate to include example images of two to three dendrites with colocalization with bassoon colocalization along the dendritic branches. Could the authors also use some postsynaptic markers such as PSD-95 or homer 1A? This is an important point since the putative synapse distribution in Figs 4 - 6 looks rather dense and ectopic expression of the FAP/YFP-neuroligin recombinant protein would result in false positive synapse counts.

## Author Response

To the Editor:

We were pleased to hear that reviewers for our recent submission to eNeuro “Fluorescence-based quantitative synapse analysis for cell-type specific connectomics” found the work potentially suitable for publication. Reviewers recognized that the method could be effective for rapid and large-scale analysis of synaptic connectivity and requested minor clarifications or additional information, which we have now included in our revised manuscript.

Below we respond to specific questions and indicate where revisions have been made in the manuscript. We have added 5 new data panels to Figure 3, and present results from extensive antibody testing for the reviewers here. We hope that this revised version will be judged suitable for publication in eNeuro. Please do not hesitate to contact me if you have any further questions.

Sincerely,

Authors

Response to specific comments

Reviewer 1

1. “...Could the authors test whether FAPpost could be imaged in vivo with two-photon microscopy?”

The possibility of using the FAPpost constructs for intravital imaging of connections in real-time is exciting and is an area of current investigation in the lab. Issues that we are addressing include MG-derivative dye selection, dose and route of administration, and customizing the optimal two photon imaging configuration for simultaneous detection and discrimination of the red dTomato fill and the far-red FAPpost synaptic marker. Pilot experiments to demonstrate the potential feasibility of this approach would not be able to systematically address these questions, nor to establish the impact (or lack thereof) of the MG in neural circuit dynamics. Thus we find that this experiment, while potentially interesting, is beyond the scope of this paper which focuses on the use of this tool in fixed brain slices.

2. The reviewer asked whether “the variability of spine density across different neurons could be due to the level of FAPpost expression,” suggesting that if weak red fluorescence was enhanced with anti-dTom immunohistochemistry we might be better able to determine whether spine variability was due to lack of detection of spines or poor cell filling.

We note that variability in spine density across putative cortical pyramidal neurons has been well-characterized in multiple prior studies, including both EM and Golgi staining (Peters and Kaiserman-Abramof, 1970; Holtmaat et al., 2005; Gray et al., 2006; Arellano et al., 2007), and appears to be a biological property of pyramidal neurons. Importantly, our analysis did not require that dTom-filled spines were detected, in order for FAPpost puncta to be assigned to a given neuron. Because neurons selected for analysis were well-isolated with respect to other labeled cells in the tissue section (see for example Figure 4-1C, we think it is unlikely that we are misassigning FAPpost puncta from another cell. Thus, variability in synapse density across cells is not coming from inadvertently misassignment (noise) in a densely labeled specimen.

Variability in puncta density could, as the reviewer suggests, come from a range of FAPpost expression levels across cells, so that weakly labeled cells might show misleadingly lower puncta densities or strongly labeled cells might show erroneous high puncta densities that do not correspond to synapses. To determine whether variability in detected puncta density was systematically related to levels of FAP/YFPpost expression, we analyzed mean puncta intensity versus detected puncta density across individual L2 Pyr neurons in our study (Figure R1). We observed no significant relationship between puncta intensity and puncta density across analyzed cells. This finding, together with the well-documented variability in spine density previously reported for cortical pyramidal neurons (Peters and Kaiserman-Abramof, 1970; Holtmaat et al., 2005; Gray et al., 2006; Arellano et al., 2007; Tjia et al., 2017), suggests that our analysis captures the real variance in synaptic density across cortical Pyr neurons.

For clarification of this point, the methods were amended on p. 8 and the following text was added on p.18:

“Analyzed cells showed no significant relationship between FAPpost puncta intensity and calculated puncta density along the dendrite.”

“It is likely that the number of viral particles infecting individual neurons was not uniform, even in cells analyzed from the same animal, and may be an additional source of heterogeneity in analysis of puncta densities; however, we found no significant relationship between puncta intensity and puncta density across analyzed neurons.”

3. The reviewer asks for further clarification regarding how FAPpost expressing neurons were included for analysis.

We have elaborated the Methods to indicate this on p. 7-8:

“Only isolated Pyr cells (typically at the edge of the viral transduction zone) that exhibited dendritic dTom as well as punctate FAP signal were selected for imaging and quantitation. FAPpost and dTom expression were not always positively correlated, an effect that was unexpected given the construct design but may be related to viral transduction time or multiplicity of infection. dTom-expressing neurons that did not exhibit membrane-localized FAP fluorescence, or showed diffuse and low-intensity signal were excluded from analysis. Analyzed cells showed no significant relationship between FAPpost puncta intensity and calculated puncta density along the dendrite.”

4. “Does FAPpost label synapses in inhibitory neurons?”

This is an interesting question that we are actively pursuing in the lab. We have observed punctate somatic and dendritic labeling in multiple subtypes of inhibitory neurons using FAP/YFPpost. For clarity and precision, we have focused our validation and input assignment analysis on pyramidal neurons. Because there are a large variety of inhibitory neuron subtypes and because these subtypes may use different combinations of neuroligins and neurexins for cell-type specific inputs, it may be necessary to validate synaptic localization independently for each molecularly-defined pre- and postsynaptic target, something that is outside of the scope of the current study.

As suggested, we have added a sentence to the results that punctate FAPpost staining is observed in inhibitory neurons, suggesting that this tool might be broadly useful for studying a variety of cell-type specific inputs on p.19:

“Although we did not systematically evaluate FAPpost properties in inhibitory neurons, we observed punctate fluorescence in PV, SST, and VIP neurons that expressed this postsynaptic label. Thus, this tool may be useful for quantitative synapse analysis in multiple cell types.”

Reviewer 2

1. “...it would be more appropriate to use example images of two to three dendrites with ... bassoon colocalization along the dendritic branches.

Synapses are extraordinarily dense in neocortical tissue, and it is challenging to accurately detect and assign immunohistochemically labeled synapses in a volume of tissue. To counter this analytical challenge, we focused on FAPpost labeling at the soma (Figure 3), since synapses here were easily detected in confocal cross-sections and the image is effectively two-dimensional.

However, we agree that analysis of dendritic synapse colocalization would enhance the validation results presented. We identified some FAPpost-labeled spiny dendritic segments (from putative Pyr neurons) and assessed FAPpost puncta for adjacent immunolabeling of bassoon at both shaft and spine synapses. We have added 5 additional panels to Figure 3, where 143 FAPpost puncta across 11 dendritic segments were analyzed for associated bassoon signal. We provide additional examples of dendritic FAPpost-bassoon alignment here (Figure R2), which could be added to the manuscript as a supplemental figure if requested.

Overall, we find that >90% of FAPpost puncta on dendrites show associated bassoon immunofluorescence.

The following text has been added on p.17:

“We also carried out bassoon immunolocalization for FAPpost puncta along spiny dendrites from putative Pyr neurons. Analysis of 10 dendritic segments showed that >90% of FAPpost puncta were associated with bassoon (Figure 3I-M). Because synapses are densely distributed across a volume of brain tissue, and there are many synapses near a labeled segment that belong to an unlabeled pair of neurons, it was not possible to determine false negative rates from these experiments (i.e. bassoon signal without corresponding FAPpost in the region of given dendritic segment). Some FAPpost puncta had no detectable bassoon associated signal. This may result from either an inability to detect bassoon (low fluorescence signal or subthreshold levels of bassoon) or from a bona fide false positive, perhaps due to FAPpost signal that may not be synaptically localized.

This is explicitly noted in the manuscript on p. 26.

“Intracellular pools of the targeting construct may contribute. Although these were digitally excluded based upon distance to the plasma membrane, this process that may not be effective for thin dendritic segments. It is possible that non-synaptic, plasma-membrane FAPpost accumulation may sometimes occur.”

2. Could the authors also use some post-synaptic markers such as PSD-95 or homer1a?”

Unfortunately, despite great effort (6 animals with 12 viral injections and multiple sections per animal), we were unable to carry out reliable immunohistochemistry for other synaptic markers such as gephyrin and PSD-95 in virally transduced brain tissue. The PSD-95 antibody (NeuroMab, 75-028, RRID: AB_10698024) immunostaining required very light fixation of acute brain slices (350μm for 10 min in 2% PFA) followed by subsectioning (40μm) for immunohistochemistry (protocol Gasser et al., 2006; antibody application reference Zhu et al., 2018). This process introduces substantial tissue loss due to fragility of the specimen. Under these conditions, only the outermost 2-3μm of the confocally-imaged section showed punctate immunofluorescence signal. Even then, the PSD-95 immunostaining was not uniform across the surface, with patchy labeling across different areas of the brain section. This may be due to uneven penetration of the fixative in the subsectioned acute brain slice. We were thus restricted to analysis of dendritic segments with both dTom fill and punctate FAPpost that happened to lie within this outermost 2-3μm that was well-labeled. Despite multiple experimental repetitions, we were unable to find unambiguous examples of clearly spiny segments (representing L2 Pyr neurons) for analysis. Although we could find dendritic segments showing FAPpost puncta colocalized with PSD-95 immunofluorescence, they are not clearly spiny and may be dendrites from inhibitory neurons. Because our analysis and validation was focused on L2 Pyr neurons, we are reluctant to more broadly examine other cell types, as targeting may differ across cell types. We show examples of FAPpost and PSD-95 association here. Note that although some FAPpost puncta colocalize with PSD-95, others do not (Figure R3). It is possible that non-colocalized puncta are inhibitory synapses; we do not expect that all FAPpost puncta will colocalize.

We also attempted to use gephyrin antibodies to examine FAPpost colocalization at putative inhibitory synapses, also in virally transduced brain tissue. We used a gephyrin antibody directly conjugated to a red (oyster550) fluorophore (Synaptic Systems), since use of the non-conjugated antibody (same clone) showed no signal under any fixation conditions, despite multiple attempts. Unfortunately, the oyster550 fluorophore is spectrally indistinguishable from dTom. We thus contracted with Synaptic Systems to make two new directly-conjugated fluorescent gephyrin antibodies using the parent clone (oyster488 and oyster633), but neither of these conjugates effectively stained brain tissue. Thus, we could only look at dendritic segments with oyster550 staining where the dTom fill was very weak and would not interfere with the immunolabel (Figure R4). Although we could observe some cases where gephyrin puncta colocalized with a subset of FAPpost puncta on a given dendritic segment (as expected if some synapses were excitatory), we are reluctant to interpret these data because of the difficulty in unambiguously separating potential dTom from gephyrin-oyster550. After many months of work, we elected to use the bassoon antibody for validation studies. We note that the neuroligin-based tag has been used in multiple other studies (Kim et al., 2011; Druckmann et al., 2013; Jun-Hyeok et al., 2018), and we hope that those published data further support the efficacy of our analytical approach.

References

Arellano JI, Espinosa A, Fairén A, Yuste R, DeFelipe J (2007) Non-synaptic dendritic spines in neocortex. Neuroscience 145:464-469.

Druckmann S, Hill S, Schürmann F, Markram H, Segev I (2013) A hierarchical structure of cortical interneuron electrical diversity revealed by automated statistical analysis. Cereb Cortex 23:2994-3006 Available at: http://www.ncbi.nlm.nih.gov/pubmed/22989582.

Gasser EMS, Straub CJ, Panzanelli P, Weinmann O, Fritschy J, Sassoe M (2006) Immunofluorescence in brain sections?: simultaneous detection of presynaptic and postsynaptic proteins in identified neurons. Nat Protoc 1:1887-1897.

Gray NW, Weimer RM, Bureau I, Svoboda K (2006) Rapid redistribution of synaptic PSD-95 in the neocortex in vivo. PLoS Biol 4:2065-2075.

Holtmaat AJGD, Trachtenberg JT, Wilbrecht L, Shepherd GM, Zhang X, Knott GW, Svoboda K (2005) Transient and persistent dendritic spines in the neocortex in vivo. Neuron 45:279-291.

Jun-Hyeok C, Su-Eon S, Ji-il K, Il CD, Jihae O, Sanghyun Y, Jaehyun L, TaeHyun K, Hyoung-Gon K, Chae-Seok L, Bong-Kiun K (2018) Interregional synaptic maps among engram cells underlie memory formation. Science (80- ) 360:430-435 Available at: https://www.ncbi.nlm.nih.gov/pubmed/29700265.

Kim J, Zhao T, Petralia RS, Yu Y, Peng H, Myers E, Magee JC (2011) mGRASP enables mapping mammalian synaptic connectivity with light microscopy. Nat Methods 9:96-102 Available at: http://dx.doi.org/10.1038/nmeth.1784.

Peters A, Kaiserman-Abramof IR (1970) The small pyramidal neuron of the rat cerebral cortex. The perikaryon, dendrites and spines. Am J Anat 127:321-355.

Tjia M, Yu X, Jammu LS, Lu J, Zuo Y (2017) Pyramidal Neurons in Different Cortical Layers Exhibit Distinct Dynamics and Plasticity of Apical Dendritic Spines. Front Neural Circuits 11:1-10 Available at: http://journal.frontiersin.org/article/10.3389/fncir.2017.00043/full.

Zhu F, Cizeron M, Qiu Z, Benavides-Piccione R, Kopanitsa M V., Skene NG, Koniaris B, DeFelipe J, Fransén E, Komiyama NH, Grant SGN (2018) Architecture of the Mouse Brain Synaptome. Neuron 99:781-799.e10.
