# Supplementary figures and images for: Fluorescence-Based Quantitative Synapse Analysis for Cell Type-Specific Connectomics
Source: eNeuro. 2019 Oct 23;6(5):ENEURO.0193-19.2019. doi: 10.1523/ENEURO.0193-19.2019 (PMC6873163; doi:10.1523/ENEURO.0193-19.2019)

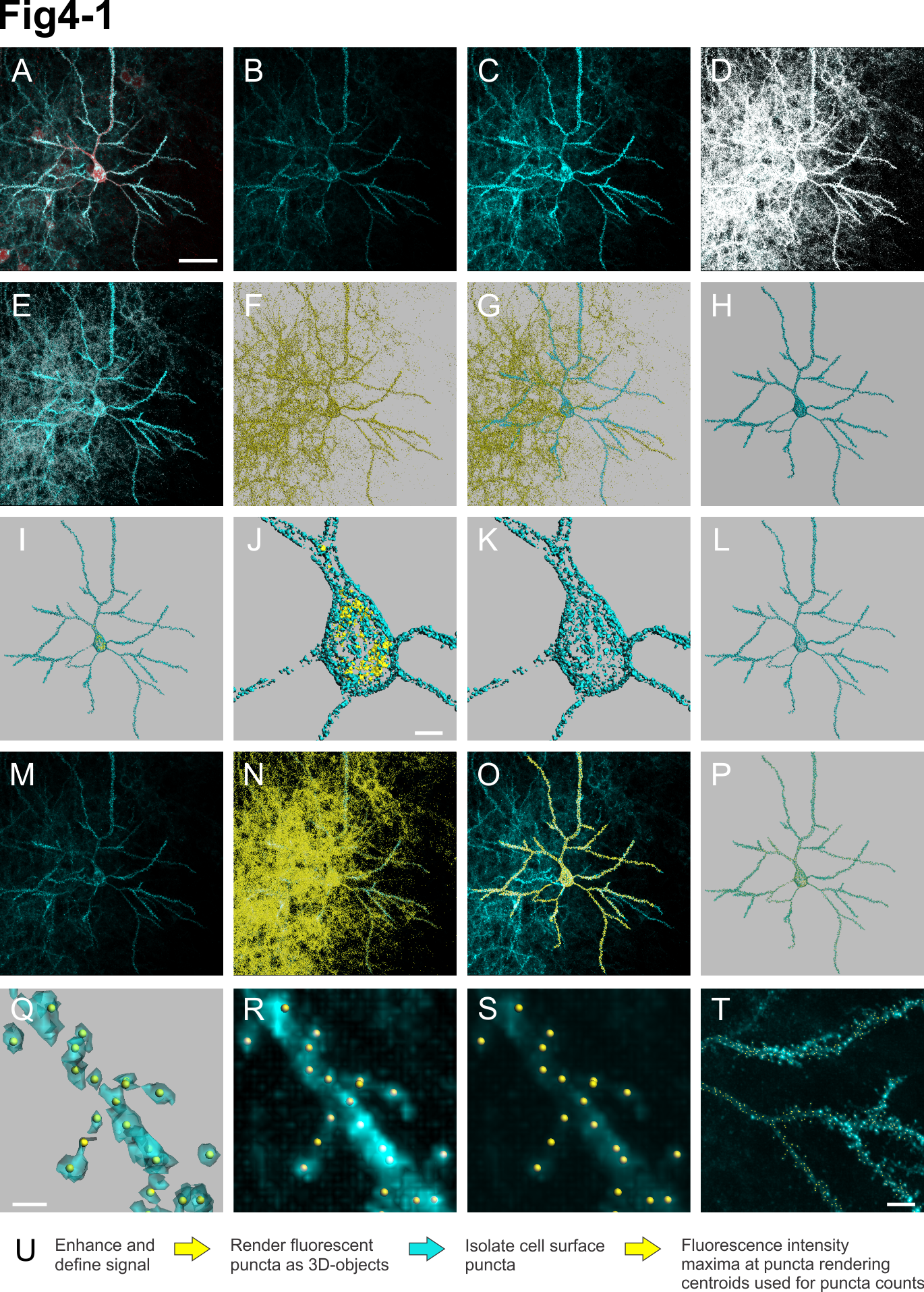

Supplement: Extended Data Figure 4-1 — YFPpost fluorescent puncta quantitation in L2/3 Pyr cell dendrites. A, Sparse YFPpost expression across the cortical column. Scale bar = 50 μm. B, L2/3 Pyr-expressing YFPpost. C, YFPpost fluorescent puncta on a dendritic shaft and spines (zoom from box in B). D, Schematic for dendritic puncta assignment (blue, assigned puncta ≤1.0 μm from shaft surface; light blue, unassigned puncta). E, 3D rendering of Pyr neuron (red) with assigned puncta. Scale bar = 20 μm. F, Dendrite from C with assigned puncta. Scale bar = 2 μm. G, Mean YFPpost puncta density for individual neurons (grey bars, ±SEM) on apical and basal dendritic branches (black dots); n = 21 cells, N = 4 animals. See also Extended Data Figure 4-2 and Table 1. Download Figure 4-1, TIF file. [file sup_enu-eN-MNT-0193-19-s02.tif]

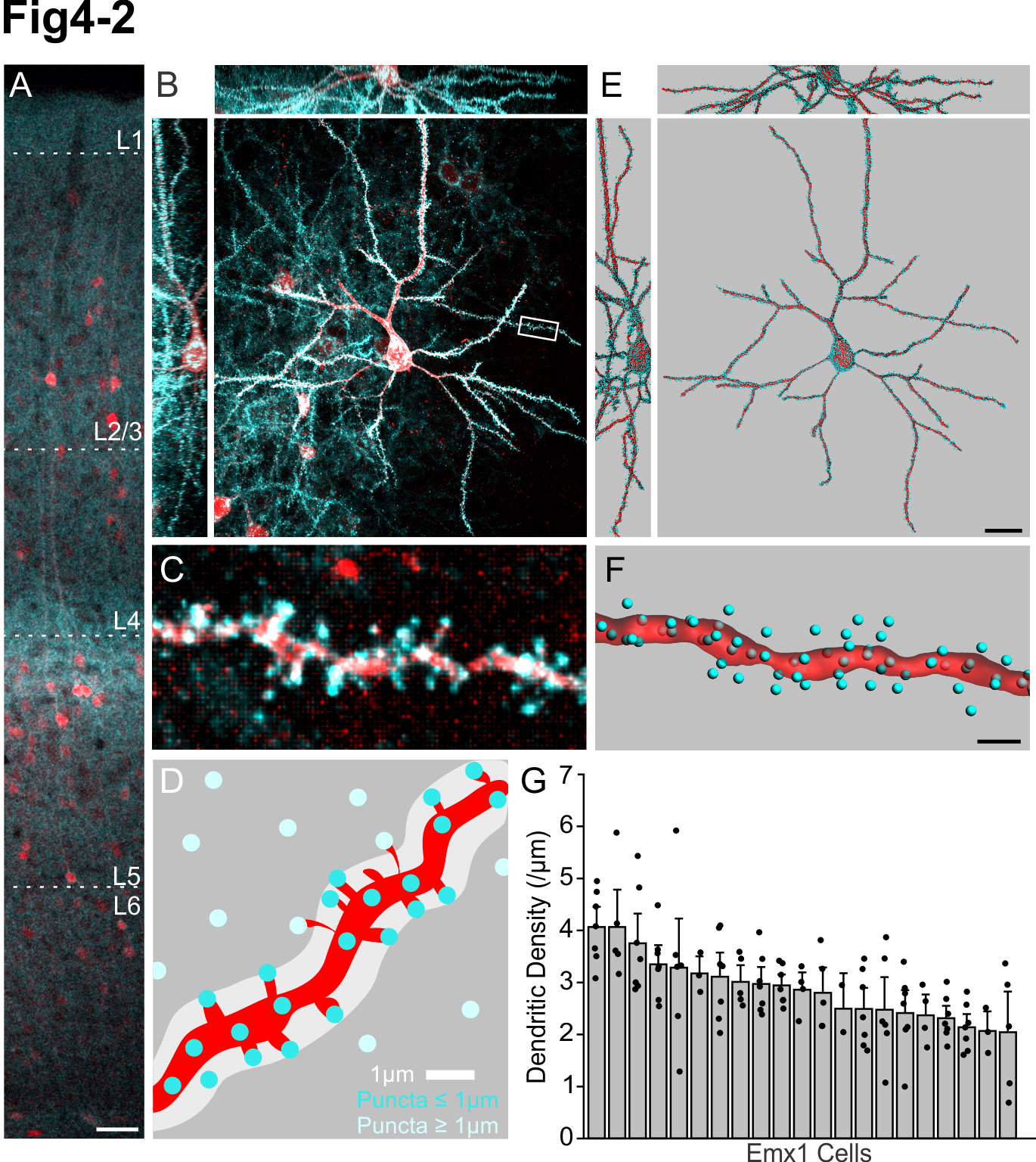

Supplement: Extended Data Figure 4-2 — Fluorescent puncta detection method using Imaris. A, Image files imported into Imaris. Scale bar = 40 μm. B, Raw synaptic fluorescent signal. C, Gain-adjusted view of synaptic fluorescent signal to visualize dim YFP signal. D, White mask of synaptic fluorescence differentiates signal from background. E, Puncta creation parameters: 0.5 μm estimated diameter, larger than three voxel size (grey pixels). F, 3D renderings of all fluorescent puncta (yellow). G, Puncta within 0.5 μm from cell surface (edge-to-edge; blue). H, Isolated cell-associated puncta (blue). I, J, Cytoplamic puncta (≤0.5 μm from cell surface; yellow). Scale bar = 5 μm. K, L, Isolated cell-surface puncta. M, Raw synaptic fluorescence used for Imaris spot detection. N, Local fluorescence intensity maxima identified using automatic detection parameters (yellow spots). O, 3D rendering of puncta centers (from L) as spots. P, Q, Alignment of puncta 3D renderings and spots. Scale bar = 1 μm. R, Alignment of spots and signal enhanced fluorescence. S, T, Alignment of spots and raw fluorescence. Scale bar = 5 μm. U, Workflow summary. Download Figure 4-2, TIF file. [file sup_enu-eN-MNT-0193-19-s03.tif]

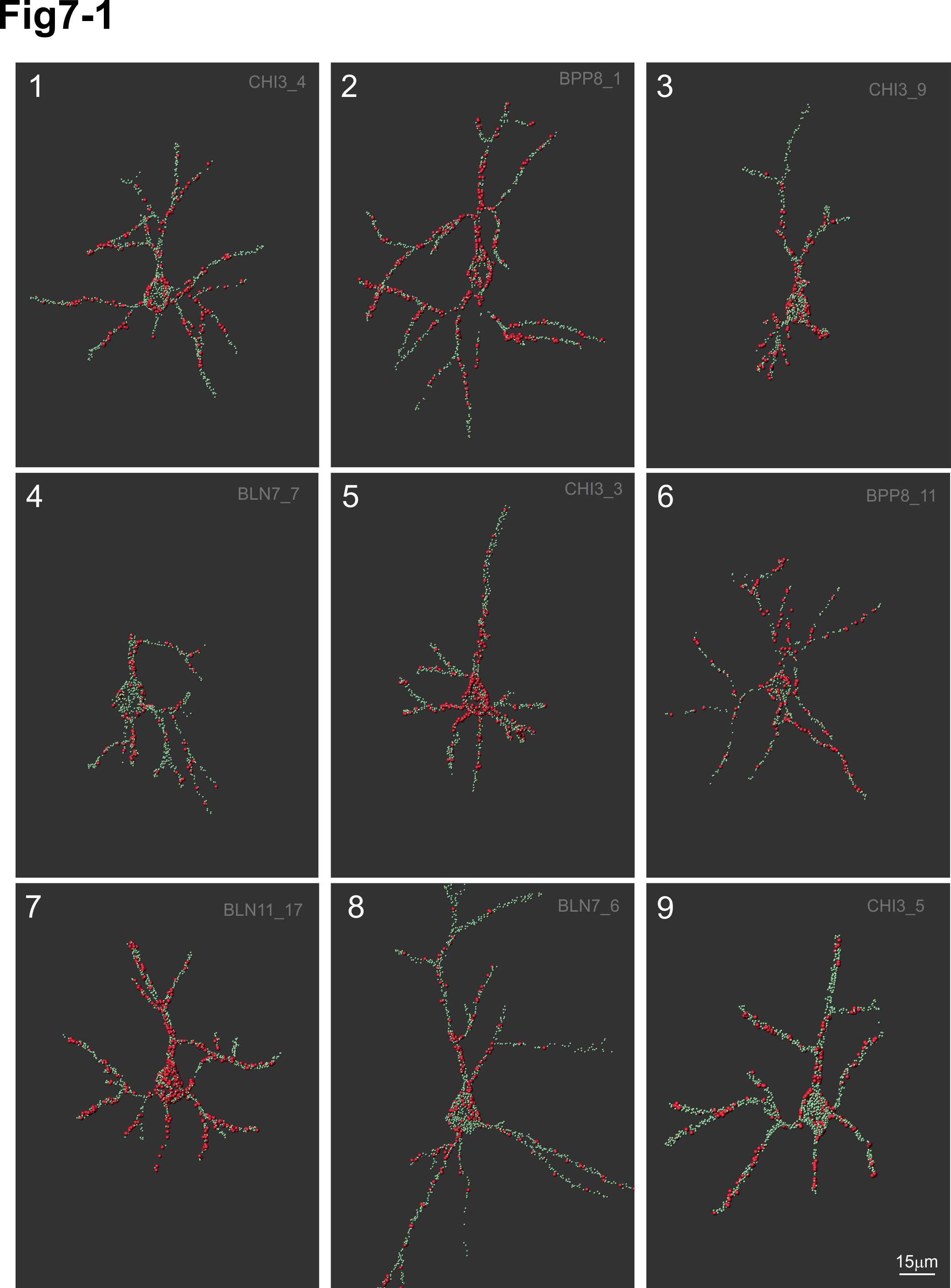

Supplement: Extended Data Figure 7-1 — PV-assigned FAPpost puncta distribution across Pyr cells. Small light-green spheres are un-assigned FAPpost puncta. Large colored spheres are input-assigned FAPpost puncta. Scale bar = 15 μm. Download Figure 7-1, TIF file. [file sup_enu-eN-MNT-0193-19-s04.tif]

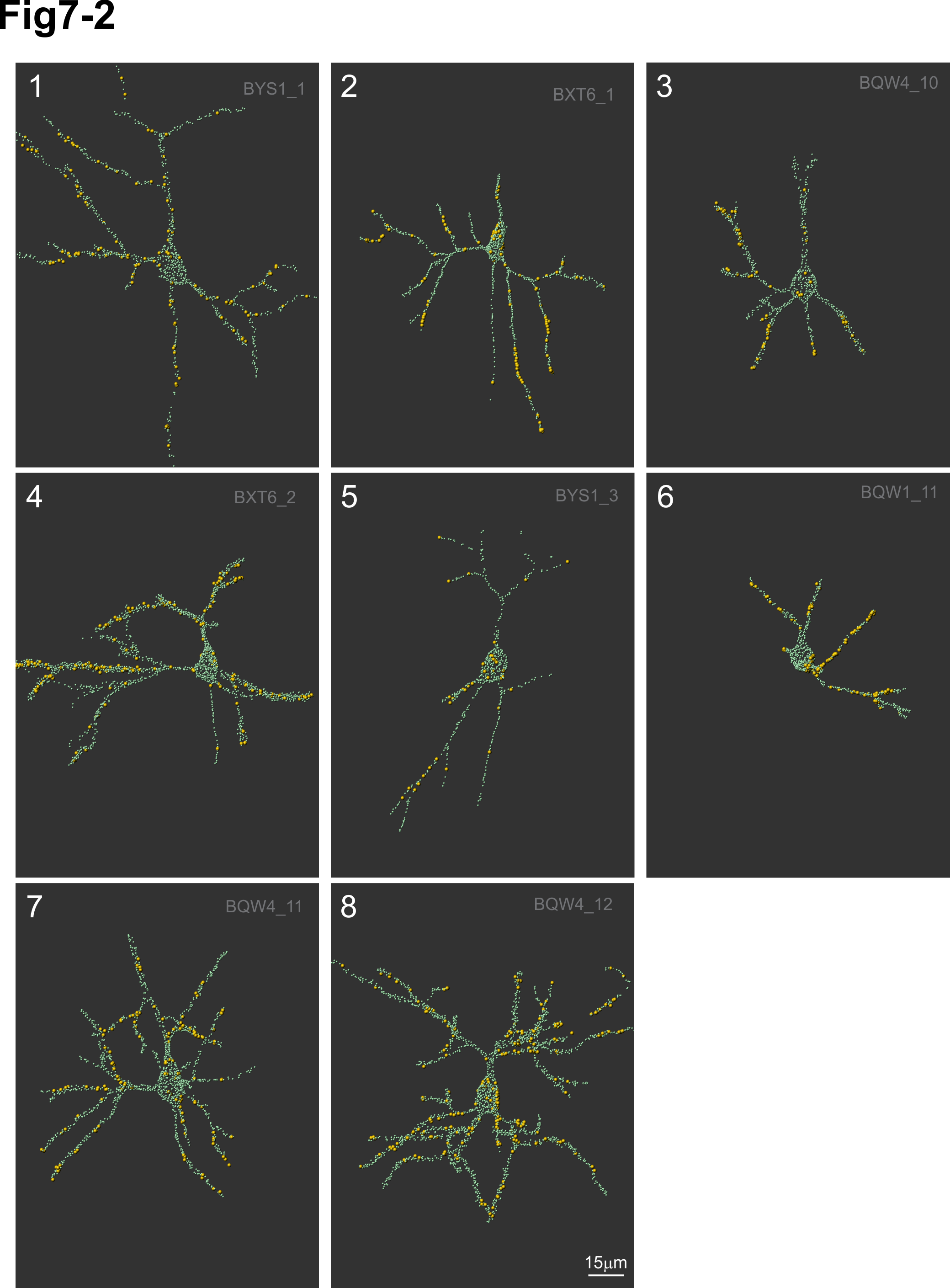

Supplement: Extended Data Figure 7-2 — SST-assigned FAPpost puncta distribution across Pyr cells. Small light-green spheres are un-assigned FAPpost puncta. Large colored spheres are input-assigned FAPpost puncta. Scale bar = 15 μm. Download Figure 7-2, TIF file. [file sup_enu-eN-MNT-0193-19-s05.tif]

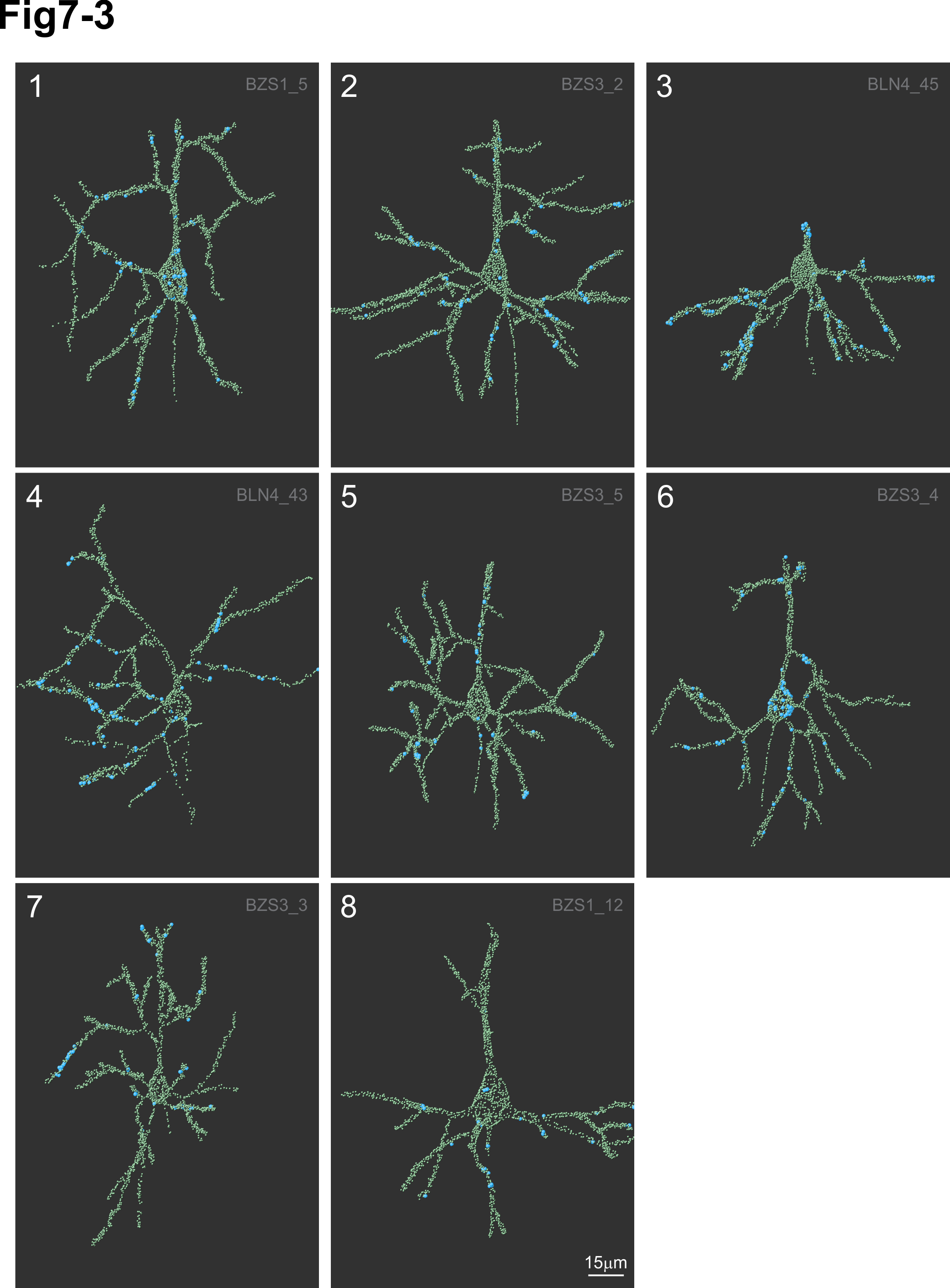

Supplement: Extended Data Figure 7-3 — VIP-assigned FAPpost puncta distribution across Pyr cells. Small light-green spheres are un-assigned FAPpost puncta. Large colored spheres are input-assigned FAPpost puncta. Scale bar = 15 μm. Download Figure 7-3, TIF file. [file sup_enu-eN-MNT-0193-19-s06.tif]
